# SMAD3 and HIF-1α orchestrate metabolic transition to aerobic glycolysis as a critical prerequisite for spontaneous reprogramming of spermatogonial stem cells

**DOI:** 10.1186/s13287-025-04541-w

**Published:** 2025-07-28

**Authors:** Yihui Cai, Jian Wen, Hongyang Liu, Rui Wei, Xiaoxiao Li, Yao Dong, Keren Cheng, Kang Zou

**Affiliations:** 1 Germline Stem Cells and Microenvironment Lab, College of Animal Science and Technology, Nanjing Agricultural University, Nanjing, 210095 China; 2 Stem Cell Research and Translation Center, Nanjing Agricultural University, Nanjing, 210095 China; 3 School of Biomedical Engineering, Hubei University of Medicine, Shiyan, Hubei 442000 China; 4 Department of Obstetrics and Gynecology, Center for Reproductive Medicine, The Fourth Affiliated Hospital of School of Medicine, and International School of Medicine, International Institutes of Medicine, Zhejiang University, Yiwu, 322000 China

**Keywords:** Aerobic glycolysis, Cell fate, Cellular plasticity, Germline-to-pluripotent transition, HIF-1A activation, Metabolic reprogramming, SMAD3 signaling

## Abstract

Spermatogonial stem cells (SSCs) possess the capacity for spontaneous reprogramming during in vitro culture, while the underlying mechanisms remain poorly understood, especially why the addition of epidermal growth factor (EGF), leukemia inhibitory factor (LIF) remarkably enhanced transition efficiency. Here we employed a multi-omics approach, integrating transcriptomics, metabolomics, and DNA methylation analyses to focus on the interplay between exogenous growth factors, metabolic pathways, and signaling cascades, particularly the role of SMAD3 in these networks. Our findings reveal that SSC reprogramming is contingent upon a metabolic shift from the tricarboxylic acid (TCA) cycle to aerobic glycolysis, modulated by fluctuating SMAD3 levels. SMAD3 downregulation activates HIF-1α, inducing aerobic glycolysis to supply energy and substrates for reprogramming. Subsequent SMAD3 reactivation promotes rapid cell proliferation, facilitating successful reprogramming. This study elucidates the pivotal role of SMAD3 in modulating glycometabolic pathways driving SSC transformation, emphasizing the necessity of aerobic glycolysis following SMAD3 fluctuations for effective reprogramming, which provides novel insights into the intricate interplay between energy metabolism and stem cell plasticity and potential applications in regenerative medicine and fertility treatments.

**Supplementary Information:**

The online version contains supplementary material available at 10.1186/s13287-025-04541-w.

## Introduction

Spermatogonial stem cells (SSCs), derived from primordial germ cells (PGCs), robustly generate sperm under the regulatory effects of the testicular niche [18]. In the last century, it was discovered that PGCs could spontaneously transition to a pluripotent state during in vitro culture [29, 34], highlighting their inherent pluripotency. Similarly, SSCs, traditionally viewed as unipotent, have been found capable of abrogating lineage commitment and spontaneously converting to pluripotent cells after long-term in vitro culture. These reprogrammed cells share many features with embryonic stem cells (ESCs) derived from the inner cell mass (ICM), including the ability to induce teratoma formation and contribute to chimeric animals [13, 35]. This unique reprogramming event distinguishes SSCs from induced pluripotent stem (iPS) cells, as it occurs without the introduction of exogenous genes or gene products, suggesting a unique potential in cellular therapy with minimal risk.

However, controlling the efficiency of SSC transformation remains challenging, as the mechanisms underlying their spontaneous reprogramming are largely unknown. Notably, the loss of *p53* remarkably accelerates SSC transformation [13], potentially linked to methylation changes mediated by DNMT1 [39]. Our recent studies have analyzed chromatin accessibility changes triggered by *p53* deletion, revealing an increase in the accessibility of the SMAD3 binding domain during the initial phase of cell transformation, alongside a notable decrease in SMAD3 protein levels. As the culture period extends, SMAD3 expression begins to rise, and the cell proliferation rate starts to accelerate, culminating in pluripotent transformation [22]. Additionally, we found that SMAD3 can directly bind to the promoter of the key pluripotency gene, *Nanog* [22]. Consistent with these findings, exogenous activation of SMAD3 in the early stage of SSC culture (germline commitment) led to cell death, whereas its activation of SMAD3 in transforming SSCs (with a pluripotent cell fate) increased transformation efficiency [22]. This oscillatory change in SMAD3 expression—initially decreasing and subsequently increasing—suggests that inhibiting SMAD3 expression in the early stage of transformation might be a prerequisite for spontaneous reprogramming of SSCs. However, the molecular underpinnings of SMAD3 fluctuations and cell fate transformation remain unclear.

A more recent study from our laboratory reported that addition of EGF/LIF to SSC culture medium significantly improved the transformation efficiency of SSCs maintained on fresh mouse embryonic fibroblast (MEF) feeder layers, particularly noting that EGF signaling activates *Smad3* expression through RAC1 during SSC transformation [44]. Moreover, transcriptomic analysis revealed significant changes in the expression of metabolism-associated genes, especially those related to carbohydrate and energy metabolism, during the initiation stage of SSC transformation [44]. Increased glycolytic activity, a hallmark of rapid proliferation in both tumor cells and stem cells, is characterized by a preference for glycolysis even in the presence of adequate oxygen—a phenomenon known as aerobic glycolysis or the Warburg effect. This dysregulation of glycolysis provides cells with essential nutrients and ATP, restructuring the microenvironment to facilitate rapid growth and metastasis by acidifying the extracellular milieu and disrupting the extracellular matrix [11, 23]. These observations imply some potential connections between SMAD3 and energy metabolism in SSCs spontaneous reprogramming.

SMAD3 is a key transcription factor downstream of classical TGF-β signaling and plays a pivotal role in the regulation of cell proliferation, apoptosis, differentiation, epithelial–mesenchymal transition, tumor suppression and cancer promotion [7, 42]. Accumulating evidence suggests that SMAD3 activity is closely associated with metabolites, for example, the reduced de novo fatty acid synthesis and increased fatty acid β-oxidation lead to intracellular acetyl-CoA accumulation, which promotes SMAD3 acetylation and entry into the nucleus to regulate endoderm differentiation [47]. Based on these observations, we proposed that SMAD3 might function as a molecule that connects energy metabolism and cell fate determination in the SSCs' spontaneous reprogramming.

Notably, the shift from TCA to aerobic glycolysis entails a reprogramming of glucometabolic gene expression, orchestrated by Hypoxia-inducible factor (HIF)-1α, a transcription factor with a helix-loop-helix structure that is activated in response to hypoxia and homeostasis. HIF-1α directly or indirectly regulates the expression of several genes involved in cell differentiation, including *Ldha* (lactate dehydrogenase-A), *Vegf* (vascular endothelial growth factor), *Flt1* (FMS-associated tyrosine kinase 1), *Pdgf-β* (platelet-derived growth factor-β), *bFgf* (basic fibroblast growth factor), and other genes that affect glycolysis [17, 33], suggesting that it plays a role in the regulation of glycolytic activity. More importantly, HIF-1α can bind to the MH2 domain of phosphorylated SMAD3 and switch the function of TGF-β to promoting glycolysis under hypoxic conditions by altering its SMAD binding partner [10]. In addition, in tumor cells, increasing evidence has revealed the connections among HIF-1α, TGF-β/SMAD3 signaling and glycolysis: TGF-β signaling inhibits glycolysis under normoxic conditions but significantly promotes glycolysis under hypoxic conditions in tumor cells in vitro and in vivo, and SMAD3 plays a key role in the metabolic reprogramming associated with tumor progression, including tumor glycolysis [46], glutaminolysis [2], and lipid metabolism [20]. These findings imply that HIF-1α plays a role in SMAD3-mediated energy metabolism reprogramming during SSC transformation.

In this study, we aim to delineate the cooperative roles of SMAD3 and HIF-1α in driving metabolic reprogramming toward aerobic glycolysis and to establish this transition as a prerequisite for the spontaneous pluripotency acquisition in SSCs. By integrating transcriptomic, metabolomic, and functional analyses, we further elucidate how metabolic-epigenetic crosstalk orchestrates SSC fate determination. Our study provides new insights into the mechanism of spontaneous reprogramming of SSCs, offering valuable information for the treatment of reproductive diseases and for future advancements in stem cell research and animal cloning technology.

## Results

### Induction of SSC pluripotency via an optimized culture system

**Fig. 1:**
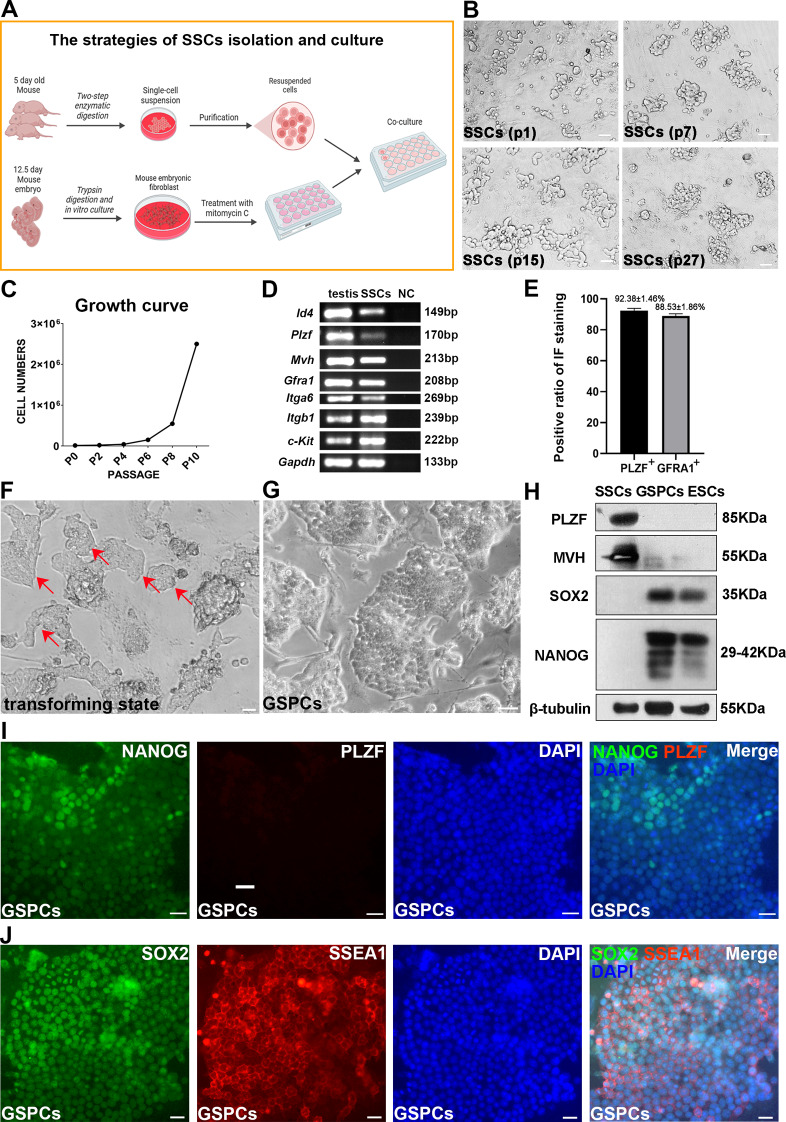
Characterization and Pluripotent Transition of Spermatogonial Stem Cells (SSCs). (**A**) Schematic of SSC isolation and culture on mouse embryonic fibroblast (MEF) feeder layer. (**B**) Clonal morphology across different passages of SSCs. (**C**) Growth kinetics of SSCs. (**D**) Identification of SSC-specific markers by RT-PCR. (**E**) Quantitative analysis of the proportion of PLZF + and GFRA1 + cells. (**F**) Observation of a transforming state in SSCs during prolonged culture. (**G**) Representative colonies of transformed GSPCs from long-term SSC cultures. (**H**) Assessment of germline and pluripotency marker expression by Western blot. (**I**, **J**) IF assays revealing pluripotency marker expression in GSPCs (**I**: NANOG, PLZF, DAPI, merged; **J**: SOX2, SSEA1, DAPI, merged). Data are presented as mean ± SD (*p < 0.05; **p < 0.01). Scale bars, 20 μm

To induce spontaneous reprogramming, a long-term cultured SSCs cell line was established. SSCs, isolated from the testes of neonatal mice, were purified and cultured on MEFs for 4–5 days (Fig. 1A). The resulting colonies exhibited a grape-like cluster morphology, characteristic of SSCs, and could be stably maintained on a MEF feeder layer for more than 30 passages (Fig. 1B), exhibiting a robust proliferation rate throughout the culture process (Fig. 1C). To confirm that the obtained cells were SSCs, total RNA was extracted and subjected to RT‒PCR analysis, which revealed the expression of key germline markers, including *Id4*, *Plzf*,* Mvh*, *Gfrα1*, *Itgα6*, *Itgβ1*, and *c-Kit* (Fig. 1D). Immunofluorescence (IF) assays further confirmed the expression of germ cell marker MVH, undifferentiated spermatogonia marker PLZF, and SSCs marker ID4 (Figure S1). The percentages of PLZF-positive and GFRA1-positive cells were 92.38 ± 1.46% and 88.53 ± 1.86%, respectively (Fig. 1E), suggesting that SSCs were efficiently enriched in the obtained cells.

As described in a previous study [44], SSCs cultured on fresh MEF feeder layers for approximately 5–6 generations could be transformed into pluripotent state cells after 2–3 generations exposed to 10 ng/mL LIF and 20 ng/mL EGF (Fig. 1F). These cells were identified as being in an intermediate state of transformation [44]. After sustained culture for an additional 5 generations, all colonies adopted an epithelium-like phenotype (Fig. 1G). These transformed cells highly expressed the pluripotency markers SOX2, OCT4, and NANOG but expressed very low levels of the germ cell marker MVH and the SSC marker PLZF (Fig. 1H). IF assays corroborated these findings, revealing nearly ubiquitous expression of NANOG, SOX2 and SSEA1 but no expression of PLZF (Fig. 1I and J). In summary, these data indicate that SSCs undergo a transition to a pluripotent state upon the addition of LIF and EGF; these cells are termed germline stem cell-derived pluripotent stem cells (GSPCs) [44]. The proportion of GSPCs progressively increased with continued culture and completed transformation after approximately 5 passages, accompanying with metabolic changes [44], while the mechanism remained unclear.

### Metabolic reprogramming and changes in energy flux during the transformation of SSCs to GSPCs

**Fig. 2:**
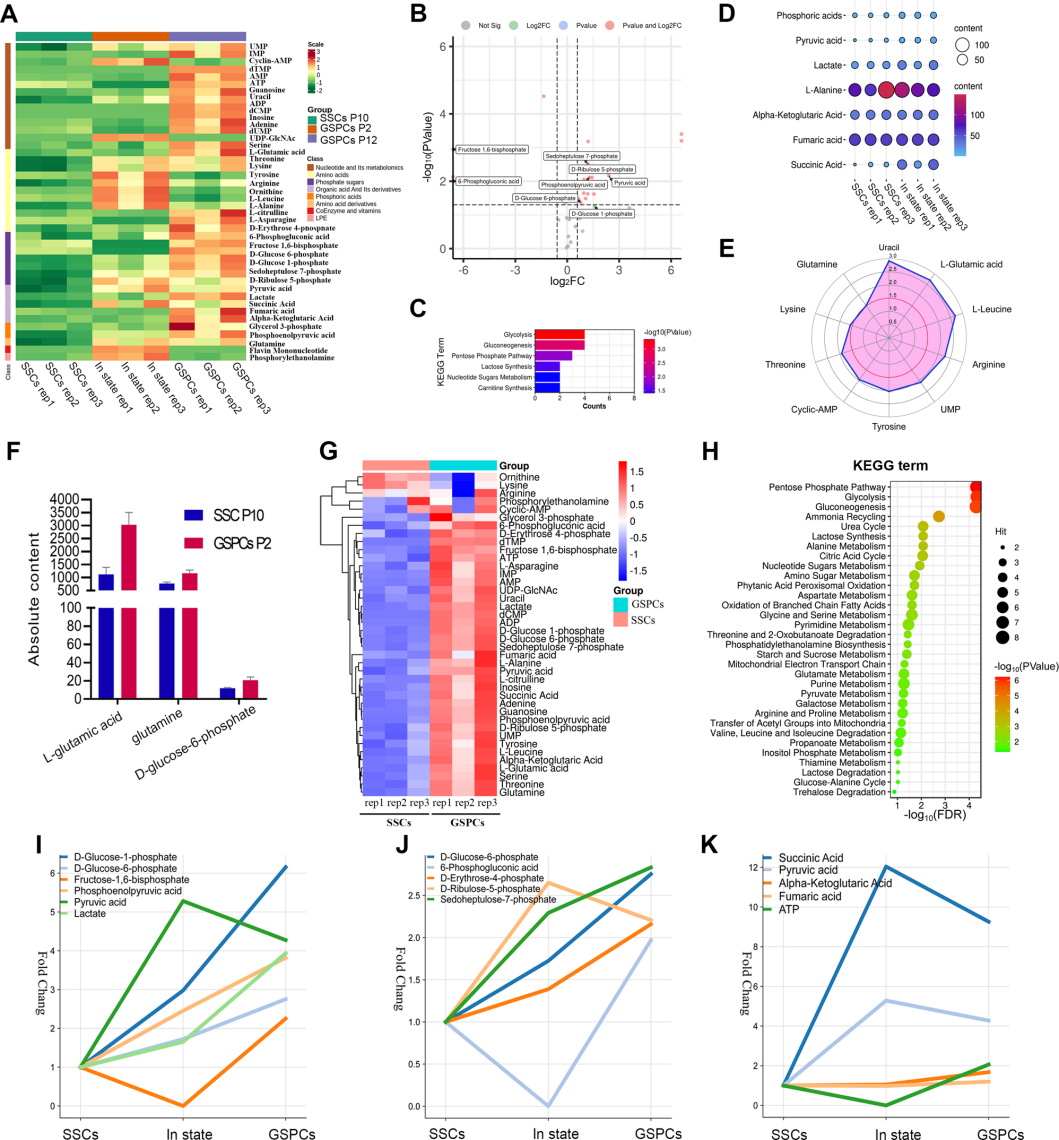
Metabolic reprogramming dynamics observed during SSC transformation. (**A**) Sample cluster diagram. (**B**) Volcanic map of SSC p10 vs. GSPCs p2 differential metabolites. (**C**) KEGG enrichment analysis of upregulated metabolites. (**D**) Bubble chart of changes in the content of pyruvate and downstream metabolites. (**E**) Radar chart of changes in the content of some amino acids and nucleotides (The values in the figure represent Fold Change). (**F**) Comparison of L-Glutamic acid and glutamine content with D-Glucose-6-phosphate content. (**G**) Cluster analysis of metabolites in SSC p10 and GSPCs p12. (**H**) KEGG enrichment analysis of up-regulated metabolites in GSPCs p12. (**I-K**) Overall trends of different metabolic pathways during transformation: change trend of metabolites involved in glycolysis pathway (**I**), change trend of metabolites involved in PPP (**J**), and change trend of metabolites involved in TCA (**K**). The metabolites displayed in this figure were specifically selected based on their significantly elevated expression profiles observed in GSPCs through targeted metabolomics screening

To explore the role of metabolic changes during SSC transformation, especially the connection of transcriptomic alterations such as Ras/Rac1/p38, TGF-β and JAK-STAT signaling pathways and energy metabolisms, we harvested stable cultured SSCs (p10 in culture medium), intermediate-state cells (p10 SSCs cultured in transformation medium for 2 passages, referred to as In state cells), and stable GSPCs (p10 SSCs cultured in transformation medium for 12 passages) for metabolomics analysis (Figure S2A). In total, 42 metabolites involved in energy metabolism were detected by using LC‒MS/MS and a self-built metabolic database from the Maiwei Company. The results were subjected to principal component analysis (PCA) and cluster analysis (Figure S2B), which revealed the pattern of differences in metabolite abundance among the 3 groups (Fig. 2A, Table S3).

### Metabolic changes during the initial stage of SSC pluripotent transformation

Previous study revealed that after two passages in the presence of EGF/LIF, SSCs enter the initial stage of the transition to a pluripotent state, probably accompanying with metabolic reprogramming, especially changes in energy metabolism, as a critical event in reprogramming. Therefore, we compared and analyzed the altered metabolic patterns in SSCs and In state cells. Metabolomic analysis revealed 25 differentially abundant metabolites between SSCs and In state cells, of which 20 showed increased abundance (*p* ≤ 0.05, fold change ≥ 1.5) and 5 showed decreased abundance (*p* ≤ 0.05, fold change ≤ 0.5) (Fig. 2B). The upregulated metabolites included glucose 6-phosphate, phosphoenolpyruvic acid, pyruvate, D-5-ribulose phosphate, and 7-sedoheptulose phosphate. We performed metabolic pathway enrichment analysis of the 20 metabolites with increased abundance by using the SMPDB database and MetaboAnalyst 5.0, and a total of 43 metabolic pathways were enriched. There were 6 pathways for which P was ≤ 0.05: glycolysis, gluconeogenesis, the pentose phosphate pathway, lactose synthesis, carnitine synthesis and ribonucleotide metabolism (Fig. 2C). This indicates that during the early stage of transformation, the glucose metabolism patterns of the cells changed significantly, and the activities of the glycolysis and pentose phosphate pathways were markedly increased. ATP, $\:\beta\:$-D-fructofuranose 1,6-bisphosphate, 6-phospho-D-gluconic acid, L-citrulline and adenine exhibited decreased abundance (Fig. 2A, Table S3). The observed decrease in the concentration of ATP, the main intracellular energy carrier, further proves that an increase in glycolytic activity leads to a decrease in the efficiency of intracellular energy production.

We noted a substantial increase in pyruvate concentrations, with a fold change of 5.28, during the initial phase of SSC pluripotent transformation (Table S3). Given the critical role of pyruvate in maintaining stem cell pluripotency [32], we examined alterations in the downstream metabolic products of pyruvate (Fig. 2D). Alanine, which can be directly synthesized from pyruvate via a transamination reaction, displayed a negligible fold change of 0.97 (Table S3). Although pyruvate predominantly enters the mitochondrial TCA cycle under aerobic conditions, only the succinate level was significantly upregulated, with a fold change of 12.04. Neither α-ketoglutarate (fold change = 1.04) nor citrate (fold change = 0.98) exhibited notable changes in abundance (Table S3). This observation aligns with prior studies indicating a reduction in the protein level of dihydrolipoyl transacetylase within the pyruvate dehydrogenase complex in GSPCs. Lactate, an anaerobic byproduct of pyruvate metabolism, exhibited a fold change of 1.65, but this change was not statistically significant (*p* value = 0.12) (Table S3). Moreover, phosphoenolpyruvate, a key intermediate in gluconeogenesis, exhibited a fold change of 2.45 (Table S3). Metabolomic analyses revealed augmented nucleotide and amino acid biosynthesis activity, necessitating precursor availability and reducing flux through both the glycolysis and pentose phosphate pathways (Fig. 2E). These findings suggest that the increase in pyruvate abundance, attributed to enhanced glycolytic flux, does not contribute to oxidative metabolism via the TCA cycle but rather facilitates glucose homeostasis through gluconeogenesis and lactate clearance.

Subsequently, we checked on the concentration of glutamine during the early dedifferentiation stages of SSCs, since it’s essential for amino acids, nucleotides, and fatty acids and intracellular protein synthesis [28], as well as stemness maintenance [51]. In the *In state* cells, glutamine and glutamate exhibited the highest absolute concentrations among all assessed metabolites, which were 146-fold and 56-fold greater than that of 6-phosphogluconate, respectively. The fold changes in glutamine and glutamate relative to the baseline levels were 1.5 and 2.7, respectively (Fig. 2F). These data indicate an increased cellular preference for glutamine during the initial stages of pluripotent transformation, facilitating increased biosynthetic activity within the cell.

### Metabolic changes during SSC-to-GSPC transformation

Following the addition of growth factors and subsequent culture through the 12th generation, the SSCs transitioned to pluripotent stem cells. Thus, the associated changes in metabolic patterns reflect the metabolic preferences of pluripotent cells, providing insights into the metabolic reprogramming underlying pluripotent transformation. Metabolomic analysis revealed that a total of 32 metabolites increased in abundance (*p* ≤ 0.05, fold change ≥ 1.5) during the transition from SSCs to GSPCs, while no metabolites that decreased in abundance (*p* ≤ 0.05, fold change ≤ 0.5) were detected (Fig. 2G). This indicates that overall metabolic activity might be enhanced or that the degradation of these molecules might be inhibited in GSPCs. Enrichment analysis of metabolic pathways revealed 32 pathways for which p was ≤ 0.05 (Fig. 2H), including the pentose phosphate pathway, glycolysis/gluconeogenesis, the tricarboxylic acid cycle, amino acid metabolism, purine/pyrimidine metabolism, and fatty acid metabolism. Collectively, these pathways cover the major metabolic routes within cells, indicating a broad increase in the metabolic activity of pluripotent GSPCs post-transformation.

To further investigate the differences among the metabolic profiles of SSCs, In state cells and GSPCs, we conducted a comparative analysis of predefined metabolites in glycolysis, the pentose phosphate pathway, and the TCA cycle, selected based on their pathway centrality and significant changes (*p* ≤ 0.05, fold change ≥ 1.5). These metabolites were prioritized for their established roles in pluripotency-associated metabolic remodeling (e.g., glycolysis for ATP production, PPP for nucleotide synthesis). In our investigation of the glycolysis pathway (Fig. 2I), the rate-limiting intermediate fructose 1,6-bisphosphate markedly decreased to below detectable levels in the *In state* cells, but in GSPCs at p12, it had risen to 2.25-fold higher than that of SSCs. Phosphofructokinase is the rate-limiting enzyme for the production of fructose 1,6-bisphosphate, suggesting that in the early stages of conversion, the downstream metabolic demand for fructose 1,6-bisphosphate far exceeds the supply of upstream glucose. Overall, pyruvate “the end-product of glycolysis” tended to increase, indicating enhanced glycolytic activity post-conversion. However, a significant peak in the In state cells followed by a decrease after the transition to multipotent germline cells suggested that the initial increase in glycolytic activity plays a more crucial role in the dedifferentiation of SSCs. The activity of the pentose phosphate pathway also remarkably increased throughout the transformation process (Fig. 2J), indicating robust biosynthetic activity in SSCs during the induction of pluripotency, particularly for nucleotides, amino acids, and lipid precursors, consistent with the phenotype of enhanced cell proliferation post-conversion [22]. Key intermediates 6-phosphogluconate (oxidative phase) and 5-phosphoribosyl 1-pyrophosphate (non-oxidative phase) were tracked to dissect PPP flux dynamics. In the In state cells, the concentration of 6-phosphogluconate was below the detection level, while the level of 5-phosphoribosyl 1-pyrophosphate peaked, suggesting that under the premise that there was no increase in sugar content in the culture system, there was a sudden surge in the demand for pentoses during the early stages of conversion, leading to the oxidation and decarboxylation of 6-phosphogluconate to 5-phosphoribosyl 1-pyrophosphate by 6-phosphogluconate dehydrogenase, thereby increasing the raw materials available for nucleotide and coenzyme synthesis. In our investigation of the TCA cycle (Fig. 2K), succinate (substrate-level phosphorylation node) and ATP were analyzed to evaluate oxidative metabolism plasticity. Changes in ATP levels among the three cell types corroborated the aforementioned enhancement of glycolytic activity. Due to the high demand for materials for biosynthesis during the initial stages of conversion, there was a significant reduction in the availability of compounds involved in oxidative metabolism via the TCA cycle, leading to decreased ATP synthesis efficiency. However, as GSPCs exhibit increased TCA cycle activity and shift their preference toward glutamine utilization, ATP synthesis efficiency is restored, ensuring cellular energy homeostasis. Additionally, the concentration of succinate markedly increased in the In state cells. Since the conversion of succinyl-CoA to succinate is the sole substrate-level phosphorylation reaction in the TCA cycle and generates GTP that transfers its high-energy phosphate bond to ADP to produce ATP, this indirectly shows that when glycolytic energy production is insufficient, the TCA cycle ensures ATP supply to a certain extent through enhanced substrate-level phosphorylation, maintaining the cell’s energy demand.

Following the completion of SSC transformation, glycolysis, pentose phosphate pathway, and TCA cycle activity was increased in GSPCs, revealing an overall metabolic pattern akin to “bivalent metabolism,” which is reminiscent of the pattern observed in naïve pluripotent stem cells. This phenomenon is consistent with the conclusion that energy reprogramming, especially change of glycometabolism, is closely associated with cell fate.

### Impact of glycolytic activity on the pluripotent transformation of SSCs

To elucidate the influence of metabolic flux on the pluripotent transformation of SSCs, we manipulated the glycolysis pathway by modulating extracellular glucose levels. Standard SSCs culture medium contains 58.3 mM glucose. We increased the glucose concentration to 100 mM and 150 mM over two passages, anticipating an increase in glycolytic enzyme expression. Paradoxically, our results revealed downregulated expression of key glycolytic enzymes at the transcript level (Figure S2C). Notably, GLUT4 expression decreased markedly in the presence of elevated glucose concentrations, as did the levels of hexokinase 2 (Hk2), phosphofructokinase platelet (Pfkp), and glyceraldehyde 3-phosphate dehydrogenase (Gapdh), whereas pyruvate kinase M2 (Pkm2) levels remained unaltered. This unexpected outcome suggested that an excess of glucose may overwhelm SSCs metabolism, leading to a reduction in glycolytic activity. Further investigations into the consequences of increased glucose concentrations on cell fate determinants (Figure S2D) revealed a pronounced decrease in the expression of the SSCs marker *Plzf* and the germ cell marker *Mvh*. In addition, the levels of the pluripotency-associated markers *Nanog* and *Sox2* were significantly decreased, with *Nanog* expression being suppressed particularly strongly at 150 mM glucose. These findings indicate that elevated glucose concentrations detrimentally affect SSCs self-renewal and the transition to pluripotency.

To specifically investigate the effects of diminished glycolytic activity on SSCs, we added a 1 mM or 2.5 mM concentration of the glycolytic inhibitor 2-deoxy-D-glucose (2-DG) to the culture for 48 h. This intervention markedly reduced the expression of *Plzf*, *Nanog*, and *Sox2*, while *Mvh* expression remained unaffected (Figure S2E). These data imply that inhibition of glycolysis obstructs not only the reprogramming of SSCs but also their regression to an undifferentiated state.

Collectively, these findings underscore the need for balanced glycolytic and pentose phosphate pathway activity for the accrual of energy and biosynthetic precursors at the onset of SSC transformation. Our study corroborates the pivotal role of aerobic glycolysis in the development of pluripotency, revealing a metabolic prerequisite for cellular reprogramming.

### Investigating metabolic reprogramming during SSCs transformation through combined targeted metabolomics and conventional transcriptomics analysis

In the above analysis, we identified metabolic alterations that occur during the process of SSCs transformation. To further explore the network of factors involved in the mechanisms underlying SSCs transformation, we reanalyzed the metabolomic data in combination with previously published RNA-seq data [44], including transcriptomic data from GSPCs, In state cells, and SSCs. To elucidate the macroscopic differences among the groups, PCA was conducted on the three sets of samples (Figure S3A). The results revealed that the samples did not overlap among the groups, demonstrating the existence of differences in their transcriptomic profiles. Specifically, GSPCs exhibited patterns different from those of the other two groups, while intermediate-state cells exhibited a pattern more similar to SSCs, implying increasing divergence of transcriptional profiles during SSCs transformation. To further elucidate the differences in gene expression among the groups, pairwise comparisons were conducted for the three cell types, and the numbers of differentially expressed genes (DEGs) were visualized using volcano plots (Figure S3B). Comparisons of GSPCs with SSCs and In state cells revealed 11,761 DEGs (5,313 upregulated, 6,448 downregulated) and 11,771 DEGs (5,470 upregulated, 6,301 downregulated), respectively. In contrast, only 1,206 DEGs (194 upregulated, 1,012 downregulated) were observed between In state cells and SSCs. This substantial difference in DEG numbers underscores the divergent transcriptional profiles of GSPCs, aligning with the PCA results.

**Fig. 3:**
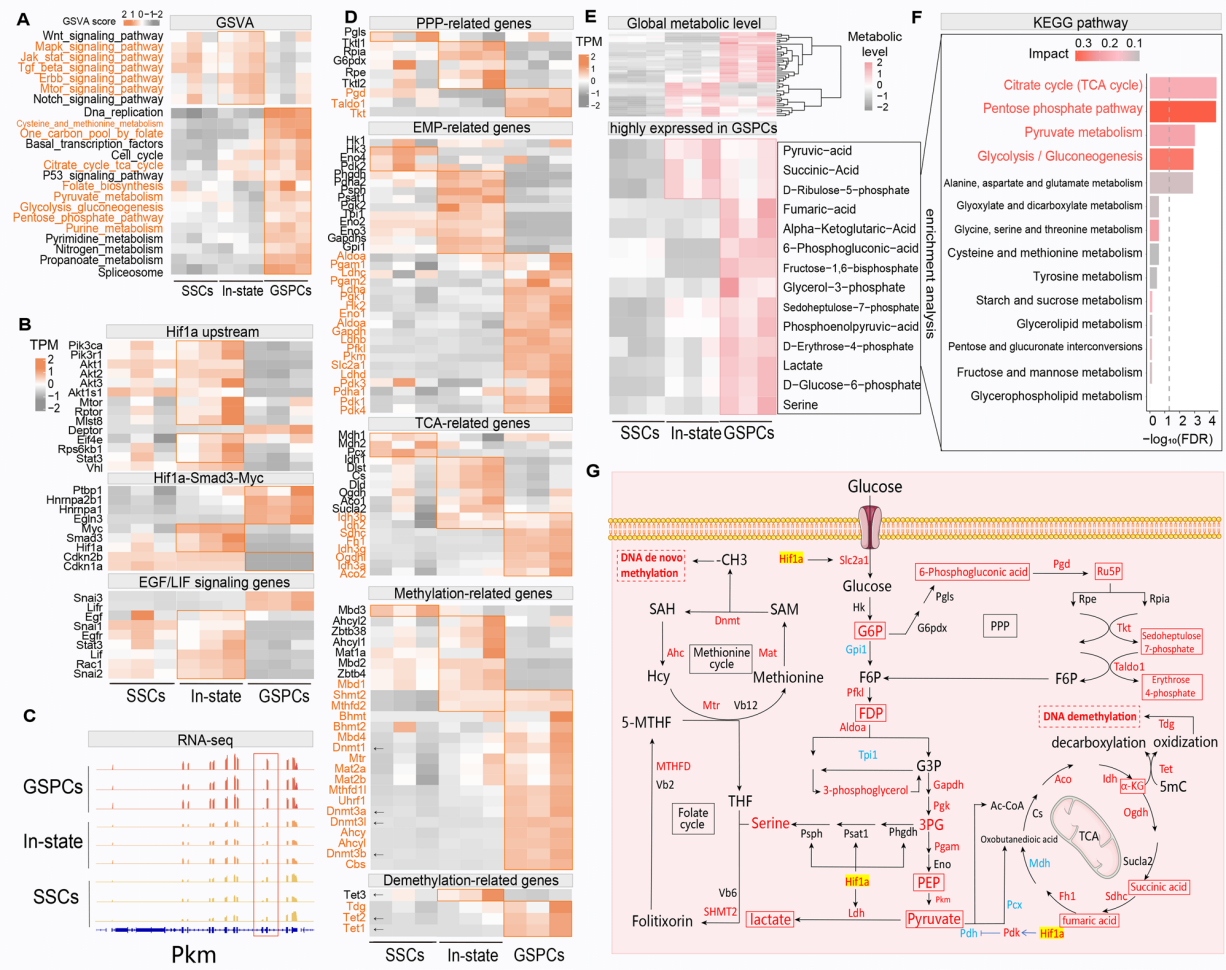
Transcriptional and Metabolic Landscape of SSCs Transformation. (**A**) GSVA analysis of RNA-seq data, with items labeled in orange indicating upstream and downstream pathways related to the Hif-1a regulatory network. (**B**) Heatmap of gene expression levels regulated by EGF and LIF in the Smad3 signaling axis(bottom). Heatmap of genes related to the Hif1a regulatory network, depicting the Hif1a-Smad3-Myc signaling axis (middle) and upstream pathways of Hif-1a (top). (**C**) Visualization in IGV of the read counts for PKM gene alignment, with the red box indicating the ninth and tenth exons of PKM. (**D**) Key genes involved in the metabolic reprogramming process of SSCs, including genes related to the pentose phosphate pathway, glycolysis pathway, TCA cycle, DNA methylation, and DNA demethylation, with genes labeled by arrows being the main genes involved in DNA methylation and demethylation. (**E**) Heatmap of expression levels of metabolites in each group, representing the overall metabolic levels of all detected metabolites (top) and the metabolic levels of highly expressed metabolites in GSPCs (bottom). (**F**) Bar plot of KEGG enrichment analysis of highly expressed metabolites in GSPCs, with items labeled in pink representing pathways related to carbohydrate metabolism, and the gray dashed line indicating the FDR threshold, with items above the dashed line having FDR < 0.05. (**G**) Summary schematic of the regulatory networks of carbohydrate metabolism, one-carbon metabolism, DNA methylation, and demethylation in GSPCs, with red indicating metabolites and enzymes highly expressed in GSPCs, blue indicating low expression in GSPCs, and highlighted yellow indicating Hif-1α-regulated targets. The metabolites highlighted by red solid-line boxes in the figure are those highly expressed in GSPCs and corresponding to Fig. 2I-K

To investigate the functions of genes specifically expressed in GSPCs, we conducted GO functional enrichment analysis on genes with high and low specificity in GSPCs (Figure S3C). Interestingly, genes that were specifically highly expressed in GSPCs were enriched in terms such as “DNA replication” and “nuclear division” related to cell proliferation, processes such as “RNA splicing” and “RNA splicing, via transesterification reactions” associated with RNA processing modifications, and processes such as “methylation,” “DNA modification,” and “histone modification” related to epigenetic modifications. These results suggest that during the transition from SSCs to GSPCs, not only is cell proliferation induced but also epigenetic modifications and RNA alternative splicing may play crucial roles in regulating this process. To further analyze the regulatory network of signaling pathways involved in SSCs transformation, we performed GSVA on the transcriptome data (Fig. 3A). The results showed that different signaling pathways were activated at different stages of transformation. Interestingly, In state cells mainly showed activation of upstream molecular signaling pathways, including “MAPK signaling pathway” and “JAK-STAT signaling pathway”. Activation of the ERBB signaling pathway is consistent with the regulatory mechanism proposed in our previous publication [44], and genes related to the EGF/LIF regulatory axis were mainly activated in In state cells (Fig. 3B). Notably, the MAPK, JAK-STAT, and mTOR signaling pathways were activated in the In state cells, along with Hif-1α, one of their common downstream target genes. To dissect the HIF-1α-centered regulatory axis (Fig. 3B), genes were selected based on their functional roles in upstream pathways (MAPK, mTOR, VHL) [49] regulating HIF-1α stability and downstream HIF-1α-SMAD3-MYC effectors, as these pathways are established regulators of HIF-1α stability and activity [10, 50] [24]. In contrast, GSPCs primarily showed activation of several downstream metabolic pathways, including “One carbon pool by folate” and “Cysteine and methionine metabolism”, which are related to one-carbon metabolism, as well as “Glycolysis gluconeogenesis” and “Pentose phosphate pathway”, which are related to carbohydrate metabolism (Fig. 3A). Notably, the MAPK, JAK-STAT, and mTOR signaling pathways were activated in the In state cells, along with *Hif-1α*, one of their common downstream target genes. The expression levels of HIF1A significantly increased in In state cells. As HIF1A is a crucial factor in the activation of the glycolysis pathway, once HIF1A expression is induced, glycolysis-related genes are activated in GSPCs that have completed pluripotent transformation. This finding implies that *Hif-1α* acts as a signaling bridge that mediates the transition from In state cells to GSPCs. In addition, the activation of the methionine cycle involved in one-carbon metabolism, which provides methyl groups for DNA de novo methylation, is consistent with the enrichment of methylation-related terms in GSPCs (Figure S3C). The entry “spliceosome” was also activated in GSPCs (Fig. 3A), consistent with the above analysis, indicating again the potentially important regulatory roles of DNA.

Our previous research indicated that Smad3 plays a crucial role in the transformation of SSCs [22], while another study demonstrated that HIF1A can bind to the phosphorylated MH2 domain of SMAD3, activating the expression of *Myc (*Huang et al., [10]. This activation releases proliferative signals and enhances the transcription of splicing factors, leading to the splicing of the PKM transcript into the PKM2 isoform and ultimately mediating metabolic reprogramming in tumor cells [10]. Based on the above analysis, HIF1A and SMAD3, key regulators of the process of SSCs transformation, may also have regulatory patterns similar to those of tumor cells. To confirm the activation of the *Hif-1α*-*Smad3*-*Myc* regulatory axis, we examined the changes in the expression of relevant genes during the transformation process in the RNA-seq data (Fig. 3B, middle). The results revealed that *Hif-1α*, *Smad3*, and *Myc* expression was specifically induced in In state cells, while the transcription of three splicing factors downstream of MYC, *Hnrnpa1*, *Hnrnpa2b1*, and *Ptbp1*, was specifically activated in GSPCs. Moreover, the levels of *Cdkn1a* (*p21*) and *Cdkn2b* (*p15*) were decreased in GSPCs due to negative regulation by MYC, thereby releasing signals promoting cell proliferation. In contrast, major genes in the PI3K-AKT-MTOR and JAK-STAT pathways upstream of *Hif-1α* were also activated in In state cells (Fig. 3B, top), consistent with the GSVA results. Notably, in In state cells, *Hif-1α* expression increases, promoting aerobic glycolysis. Moreover, we observed an increase in *Vhl* expression. VHL is a ubiquitin ligase capable of ubiquitinating and degrading HIF1A [49]. As transformation progresses, increased levels of the VHL protein may gradually lead to degradation of the highly expressed HIF1A protein. After transformation was complete, the mRNA levels of both *Hif-1α* and *Vhl* decreased in GSPCs. We speculate that this is because HIF1A is degraded by VHL after promoting aerobic glycolysis, resulting in a decrease in the transcription of *Hif-1α* and thereby causing a decrease in the transcription of *Vhl*.

As reported previously [50], PKM1 and PKM2, the two main alternative splicing isoforms of the PKM transcript, possess mutually exclusive exons: PKM1 contains exon 9, whereas PKM2 contains the mutually exclusive exon 10. The splicing factors HNRNPA1, HNRNPA2B1, and PTBP1 exhibit high affinity for exon 9 of PKM but do not bind to exon 10. Consequently, their activation results in an increase in the expression level of the PKM2 transcript. To validate the observed increase in PKM2 expression, we examined the number of reads mapped to exon 9 and exon 10 of the PKM gene to assess changes in the transcripts of the PKM1 and PKM2 isoforms (Fig. 3C). The results showed that the number of reads mapped to exon 10 of PKM in GSPCs was significantly greater than that in In state cells or SSCs. Conversely, the number of reads mapped to exon 9 was almost zero, indicating an increase in the expression level of PKM2 in GSPCs. The increased alternative splicing of PKM in GSPCs is also consistent with the results obtained from the pathway enrichment analysis described above (Fig. 3A, S3C). This suggests that during the transformation process, the activation of the *Hif-1α*-*Smad3*-*Myc* regulatory axis ultimately leads to the upregulation of PKM2 expression.

**Fig. 4:**
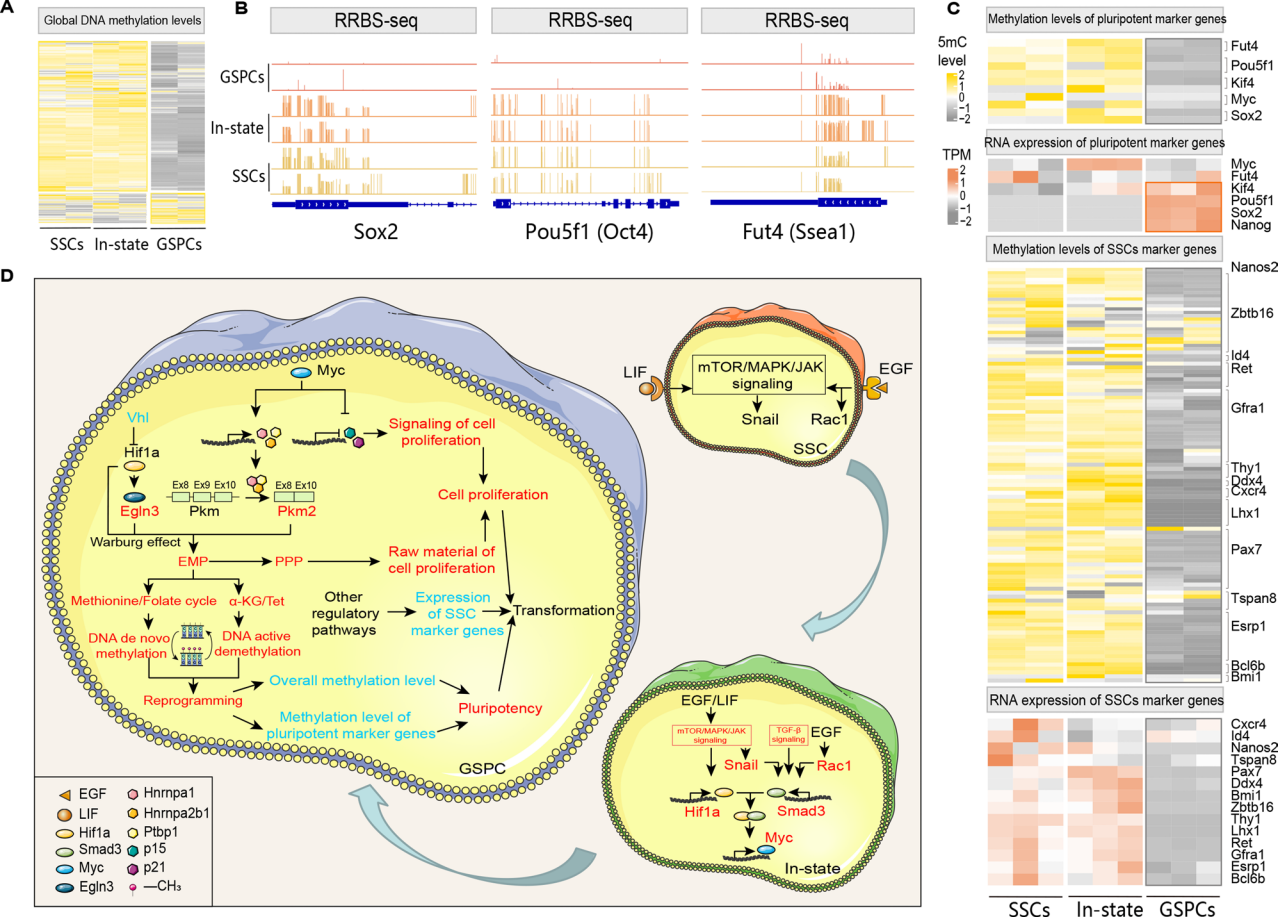
SSCs’ DNA Methylation Landscape during pluripotent transformation. (**A**) Heatmap showing overall DNA methylation levels. (**B**) IGV visualization of methylation sites in pluripotency marker genes. (**C**) Heatmaps showing expression levels and methylation levels of pluripotency marker genes and germline marker genes. (**D**) Schematic diagram of the regulatory network during SSCs differentiation. This diagram summarizes the regulatory mechanism model of SSCs’ differentiation obtained through integrated analysis of transcriptomics, metabolomics, and DNA methylation sequencing. It includes the signal preparation stage during the SSCs period, the signal activation stage during the intermediate stage, and the metabolic transition stage during the GSPCs period. Red font indicates activation during the respective stage, while blue font indicates inhibition

Studies have shown that HIF1A can interact with its downstream target proteins PKM2 and PHD3 (EGLN3), ultimately playing an important role in the reprogramming of cancer cell glucose metabolism [52]. Our previous transcriptomic analysis revealed that *Hif-1α* is highly expressed in In state cells, while *Egln3* and *PKM2* are highly expressed in GSPCs (Figs. 3B and 4C). Additionally, carbohydrate metabolism-related pathways are activated in GSPCs (Fig. 3A). We hypothesized that SSCs undergo HIF1A-mediated metabolic reprogramming during transformation. To verify this hypothesis, we conducted targeted metabolomics sequencing of SSCs, In state cells, and GSPCs and detected a total of 42 metabolites. The results showed that the detected metabolites were highly expressed mainly in In state cells and GSPCs (Fig. 3E, top), indicating that the overall metabolic activity gradually increased during the transformation of SSCs. To explore the metabolic pattern of SSCs after transformation, we identified metabolites that were highly expressed in GSPCs (Fig. 3E, bottom) and conducted KEGG pathway enrichment analysis (Fig. 3F). The results showed that the metabolites were mainly enriched in carbohydrate metabolism-related pathways such as “Citrate cycle”, “Pentose phosphate pathway”, “Pyruvate metabolism”, and “Glycolysis/Gluconeogenesis”, indicating the activation of carbohydrate metabolism pathways in GSPCs at the metabolite level, consistent with previous transcriptomic results (Fig. 3A).

To further validate the activation of glucose metabolism during SSCs transformation, we examined the changes in the expression levels of genes encoding key enzymes involved in glucose metabolism pathways (Fig. 3D). Genes in glycolysis (EMP), pentose phosphate pathway (PPP), and TCA cycle were systematically analyzed due to their pivotal roles in carbohydrate flux regulation, as evidenced by their centrality in thermodynamic-driven metabolic reprogramming [4, 30, 41]. Most key enzymes in the glycolysis pathway (EMP) and the pentose phosphate pathway (PPP) were highly expressed during the transformation process. Notably, the low expression of Gpi1 in GSPCs favors the entry of glucose-6-phosphate into the PPP rather than direct metabolism to fructose-6-phosphate. The high expression of the *Pdg* (Phosphogluconate dehydrogenase), *Tkt* (Transketolase), and *Taldo1* (Transaldolase 1) enzymes in the PPP in GSPCs (Fig. 3D), as well as the presence of the metabolites sedoheptulose 7-phosphate and erythrose 4-phosphate (Fig. 3E), also supports this observation. Activation of the PPP leads to an increase in pentose sugar supply, and pentose sugars, as one of a raw material for DNA replication, are crucial for cell proliferation, providing a material basis for the observed increase in the cell proliferation level [37], which is consistent with the results of our previous analysis (Fig. 3A, S3C). Notably, the expression levels of the HIF1A downstream targets *Slc2a1* (Solute carrier family 2, facilitated glucose transporter member 1), *Ldh* (Lactate dehydrogenase), and *Pdk* (Pyruvate dehydrogenase kinase) in the EMP [52] were also elevated in GSPCs. These compounds promote glucose uptake, increase lactate production, and inhibit the entry of pyruvate into the TCA cycle. The aforementioned changes in glucose metabolism are similar to those associated with the Warburg effect [16], indicating that SSCs undergo metabolic reprogramming similar to that of cancer cells during transformation.

In addition, our previous transcriptomic analysis indicated the activation of methylation regulation and one-carbon metabolism in GSPCs (Fig. 3A, S3C). One-carbon metabolism comprises the methionine cycle and the folate cycle, with the former providing a crucial source of DNA methylation, while the essential substrate serine in the latter can be obtained from the serine synthesis pathway (SSP) downstream of EMP [4]. Therefore, DNA methylation in GSPCs might be activated through the EMP-SSP-folate cycle-methionine cycle signaling axis. To verify this, we examined the expression levels of key enzymes and metabolites in this signaling axis. The results showed that serine is highly expressed specifically in GSPCs (Fig. 3E). *Shmt* (Serine hydroxymethyltransferase) and *Mthfd* (Methylenetetrahydrofolate dehydrogenase), key enzymes in the folate pathway, and *Ahcy* (S-adenosylhomocysteine hydrolase), *Mtr* (5-methyltetrahydrofolate-homocysteine methyltransferase), *Mat* (Methionine adenosyltransferase), and *Dnmt* (DNA methyltransferase), key enzymes in the methionine cycle, are highly expressed in GSPCs (Fig. 3D), indicating activation of the folate pathway and the methionine cycle in GSPCs. Importantly, the key enzymes responsible for de novo DNA methylation, *Dnmt3a*, *Dnmt3b*, and *Dnmt3l* [30], were highly expressed in GSPCs (Fig. 3D), suggesting that there are significant changes in DNA methylation sites between GSPCs and SSCs and implying that substantial changes in the DNA methylation landscape occur during the transformation of SSCs.

Moreover, we focused on changes in DNA demethylation during the transformation of SSCs. DNA demethylation is mediated by members of the ten-eleven translocation (TET) family and thymidine DNA glycosylase (TDG), with TET enzymes oxidizing methyl groups and TDG removing oxidized methyl groups [41]. During the process of methyl oxidation, α-ketoglutarate (α-KG), which is also an important intermediate in the TCA cycle, plays a crucial role as a substrate. By combining metabolomic and transcriptomic data, we found that α-KG, *Tet1*, *Tet2*, and *Tdg* were highly expressed in GSPCs (Fig. 3D), indicating the activation of DNA demethylation in GSPCs.

In summary, by combining metabolomic and transcriptomic data, we summarized the metabolism-regulating transcriptional network involved in the transformation of SSCs and highlighted metabolites highly expressed in GSPCs with red boxes (Fig. 3G). We found that in GSPCs, HIF1A-mediated EMP is activated, and downstream one-carbon metabolism is also activated, leading to an increase in de novo DNA methylation in GSPCs. In addition, the upregulation of *α-KG*, *Tet*, and *Tdg* in GSPCs leads to an increase in DNA demethylation levels, implying that reprogramming of SSCs occurs during the transformation process.

### Analysis of DNA methylation patterns during SSCs transformation using RRBS-seq

Through transcriptomic and metabolomic analyses, we observed that both the levels of DNA methylation and demethylation activities are increased in SSCs during the transformation process, suggesting dynamic regulatory shifts. Therefore, we performed RRBS methylation sequencing on SSCs, In state cells, and GSPCs. PCA on the methylation data revealed good repeatability and significant methylation differences among the groups (Figure S4A). Furthermore, a heatmap of the overall DNA methylation levels indicated that GSPCs have many regions of low methylation, while the methylation regions of In state cells and SSCs are similar (Fig. 4A). This suggests that after SSCs transform into GSPCs, the cellular genome transitions from a highly methylated state to a low-methylation state, resulting in a significant change in the DNA methylation profile.

To identify the locations of differentially methylated CpG sites (DMCs), we annotated gene elements and CpG regions during the transformation of SSCs (Figure S4B). Regarding gene elements, during the transformation of SSCs, there were few changes in the proportions of DMCs in promoters and exons, but significant changes were observed in introns and intergenic regions. During the transition from SSCs to In state cells, DMCs in intergenic regions (41%) dominated, while during the transition from In state cells to GSPCs, there was a significant increase in the proportion of DMCs in introns (35%), which was equivalent to that in intergenic regions (38%). During the process of transformation from SSCs to GSPCs, the proportion of DMCs in introns (36%) surpassed that in intergenic regions (28%) and became dominant. Regarding CpG sites, during the transformation of SSCs, there were significant changes in the proportions of DMCs in CpG islands, while the changes in CpG shores were relatively small. The above changes in DNA methylation regulatory patterns may play an important role in the transformation of SSCs.

In the above transcriptomic and metabolomic analyses, we found that SSCs undergo metabolic reprogramming during transformation into GSPCs, resulting in the activation of de novo methylation and demethylation processes in GSPCs. RRBS sequencing revealed a significant decrease in DNA methylation levels in GSPCs. Since promoter methylation levels are often negatively correlated with gene expression levels, we examined the methylation levels of the promoters of typical pluripotency marker genes (*Klf4*, *Sox2*, *Nanog*, *Oct4*, *Myc*, and *Ssea1*) (Fig. 4B, S4C). The results showed that all pluripotency marker gene promoters were hypomethylated in GSPCs, while the promoters of *Sox2*, *Oct4*, and *Ssea1* were hypermethylated in SSCs and In state cells. Specifically, in comparison to In state cells, the methylation levels of *Sox2* (meth.diff= -48.83, *q* < 0.01), *Oct4* (meth.diff = -43.12, *q* < 0.01), and *Ssea1* (meth.diff = -33.17, *q* < 0.01) are significantly downregulated in GSPCs (Table S7). Furthermore, when compared to SSCs, the methylation levels of *Sox2* (meth.diff = -43.97, *q* < 0.01), *Oct4* (meth.diff = -46.09, *q* < 0.01), and *Ssea1* (meth.diff = -43.31, *q* < 0.01) were also notably decreased in GSPCs. (Table S8), suggesting that the acquisition of pluripotency in GSPCs may primarily involve demethylation of the *Sox2*, *Oct4*, and *Ssea1* genes. To further validate the increased pluripotency of GSPCs, we analyzed the overall methylation levels and expression levels of pluripotency and SSCs marker genes together (Fig. 4C). The results showed that pluripotency marker genes were highly expressed and hypomethylated in GSPCs. Interestingly, SSCs marker genes were expressed at low levels and hypomethylated in GSPCs, indicating that the high expression of pluripotency genes in GSPCs may be due to demethylation, and the low expression of SSCs marker genes is not caused by methylation-induced gene silencing but by other regulatory mechanisms.

Based on the previous analysis, we proposed a model to explain the rapid transformation of SSCs upon the addition of EGF and LIF by integrating transcriptomic, metabolomic, and DNA methylation data (Fig. 4D). This model includes a signaling preparation phase during the SSCs period, a signaling activation phase during the In state, and a metabolic transformation phase during the GSPCs period. Specifically, the addition of EGF and LIF initiates the activation of the ERBB pathway in SSCs, and after at least 5 generations of cultivation in EGF/LIF medium, the TGF-β, mTOR, MAPK, and JAK-STAT pathways are activated. At this point, SSCs gradually transition to *In state* cells, with activation of the key downstream factors SMAD3 and HIF1A. The interaction between these two proteins induces the expression of *Myc*, an important pluripotency factor that also promotes cell proliferation. Consequently, with sustained expression of Myc in *In state* cells, the cells gradually enter the GSPCs stage. On the one hand, the increased expression of *Myc* promotes the transcription of the splicing factors *Hnrnpa1*, *Hnrnpa2b1*, and *Ptbp1*, leading to the preferential splicing of the PKM precursor RNA into the PKM2 isoform. On the other hand, p15 and p21, which are inhibitors of cyclin-dependent protein kinases, are inhibited by the activation of *Myc*, releasing cell proliferation signals to GSPCs. Moreover, *Vhl*, an inhibitor of ubiquitination-mediated degradation of the HIF1A protein, is downregulated in GSPCs, enabling HIF1A to play a role in GSPCs. HIF1A interacts with its downstream target proteins EGLN3 and PKM2, triggering metabolic reprogramming in GSPCs, activating the EMP pathway, inducing the Warburg effect, and subsequently providing sufficient energy to meet cellular demands.

### The expression level and phosphorylation level of SMAD3 increased during culture of SSCs

**Fig. 5:**
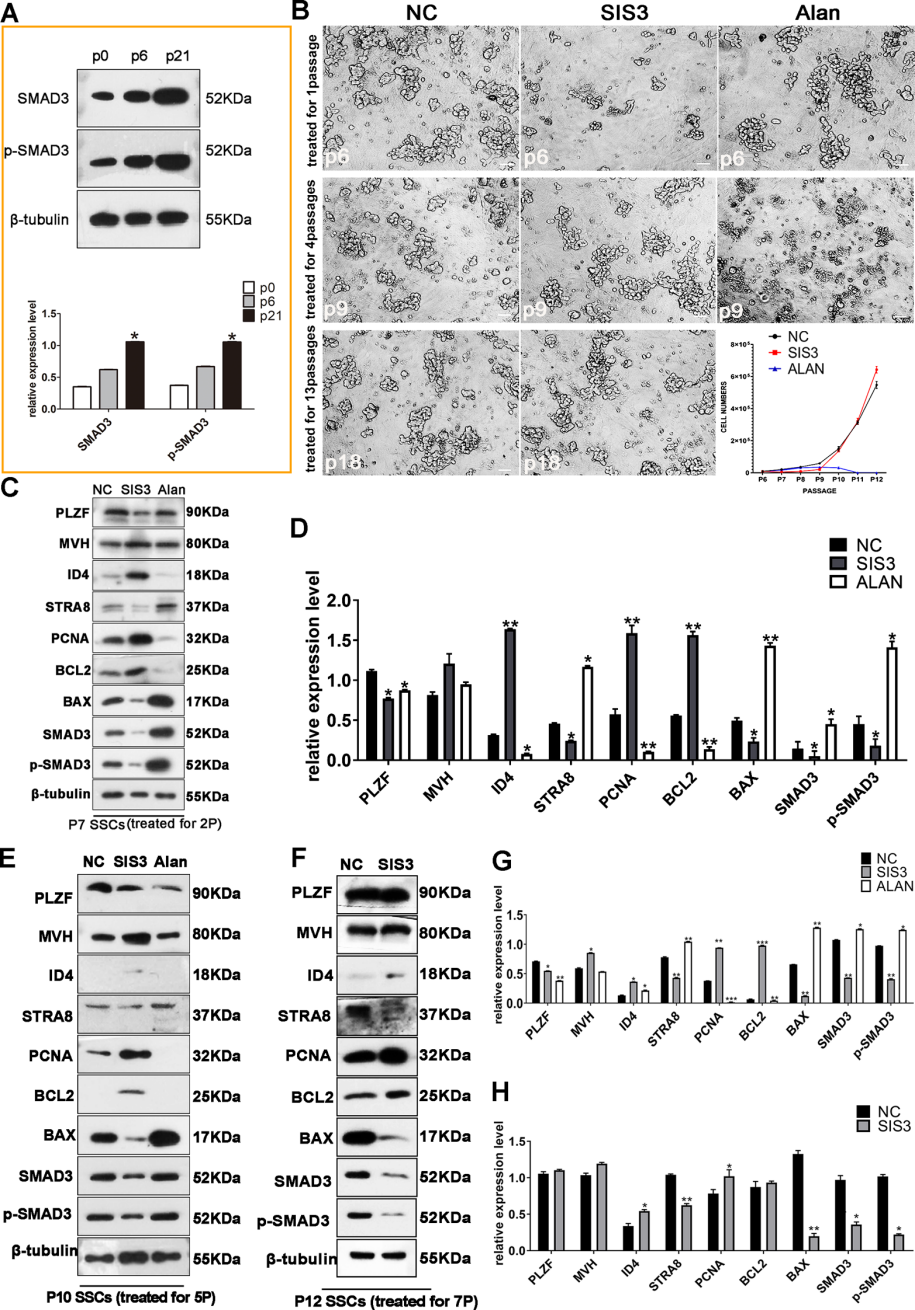
Modulation of SMAD3 Influences SSCs proliferation and differentiation. (**A**) Temporal increase in SMAD3 and phosphorylated SMAD3 (p-SMAD3) with in vitro SSCs culture duration, with statistical representation (*n* = 3, *p < 0.05, **p < 0.01). (**B**) Morphological analysis of SSCs post SIS3 or alantolactone treatment across different passages, accompanied by growth curve tracking. (**C**) Western blot detection of PLZF, MVH, STRA8, PCNA, SMAD3, and p-SMAD3 in p7 SSCs treated with SIS3 (10 µM) or alantolactone (5 µM) for two passages, with statistical analysis (*n* = 3) (**D**). (**E**) Western blot analysis of PLZF, MVH, ID4, STRA8, PCNA, BCL2, BAX, SMAD3 and p-SMAD3 in p10 SSCs treated with SIS3 or alantolactone for five passages, with statistical evaluation (*n* = 3) (**G**). (**F**) Western blot quantification of the same markers in p12 SSCs treated for 7 passages, with statistical analysis (*n* = 3) (**H**). Data are mean ± SD (*p < 0.05; **p < 0.01). Scale bars, 20 μm

Above data and analysis revealed the role of *Hif-1α*-*Smad3*-*Myc* regulatory axis in SSCs transformation, but the exact regulatory mechanisms are not clear. Subsequently, we focused on the molecular underpinning of SMAD3, especially the role in regulation of aerobic glycolysis. In *p53*-deficient SSCs, the chromatin accessibility of the SMAD3 binding domain is increased, but the expression of SMAD3 is initially downregulated during early passages and markedly upregulated during the middle to late stages of transformation, suggesting that SMAD3 may augment, rather than drive, the transformation process [22]. This is supported by our previous findings that EGF and LIF signals in turn stimulate SMAD3 expression during SSCs transformation and that this effect is concomitant with the activation of glucose metabolism-related signaling pathways at the onset of SSCs reprogramming [44]. Therefore, the observed fluctuations in SMAD3 expression prompted us to ask whether SMAD3 is associated with aerobic glycolysis during this process. To explore the function of SMAD3 in SSCs, we used SSCs that were derived from the testes of neonatal mice and maintained on a MEF feeder layer (Fig. 1B). To assess the expression and activation profiles of SMAD3 during prolonged SSCs culture, we employed Western blot analysis. Initial findings revealed that both SMAD3 and its phosphorylated form were present at low levels in newly isolated SSCs, with a marked increase following extended culture (Fig. 5A and B). Given that SSCs beyond 25 passages in our culture system exhibited an increased propensity for spontaneous reprogramming and considering that SMAD3 activation is pivotal during the intermediate and late stages of SSCs transformation after prolonged culture (approximately 20 passages) [44], we propose that the observed increases in SMAD3/p-SMAD3 expression and activity are correlated with the of SSCs passages, proliferative capacity, and reprogramming potential.

### SMAD3 plays a crucial role in maintaining the stable proliferation of SSCs

To further explore the role of changes in SMAD3 expression in SSCs transformation, we manipulated SMAD3 activity in passage 5 SSCs, which could be stably maintained in vitro without spontaneous transformation within 15 passages. After using SIS3, a selective inhibitor of SMAD3 phosphorylation that attenuates TGF-β and activin signaling [12], we observed an initial decrease in the number of SSCs. However, upon prolonged exposure to SIS3, SSCs proliferation rates not only recovered but also exceeded those of the untreated controls. In contrast, activation of SMAD3 signaling with alantolactone resulted in a progressive decrease in SSCs viability, leading to cell death after 7–8 passages (Fig. 5B). Western blot analysis after SIS3 treatment for two passages revealed reduced SMAD3 phosphorylation and decreased expression of the germ cell markers PLZF, MVH, and STRA8, with a concomitant increase in the expression of the proliferation marker PCNA (Fig. 5C and D). These data suggest that there is a selective advantage for more proliferative germ cell subpopulations under SMAD3 inhibition. Further inhibition over five passages led to increased ID4 and MVH expression, increased PCNA levels, decreased apoptosis, and a shift in the balance of BCL2 and BAX expression (Fig. 5E and G). After seven passages, a slight increase in PLZF expression was noted, and high levels of ID4 and MVH were detected, indicating sustained proliferation and reduced apoptosis (Fig. 5F and H). Conversely, activation of SMAD3 with alantolactone for two passages reduced the expression of PLZF and PCNA and increased the expression of STRA8 (Fig. 5C and D), suggesting a suppressive effect on proliferation and the induction of differentiation. After five passages, increased apoptosis was observed (Fig. 5E and G), reinforcing the notion that hyperactive SMAD3 signaling hinders SSCs proliferation and promotes differentiation. In summary, our findings indicate that SMAD3 inhibition promotes SSCs proliferation, while SMAD3 activation impedes SSCs maintenance, highlighting the pivotal role of SMAD3 in the balance between SSCs self-renewal and differentiation.

### Recovered *Smad3* expression in SSCs is required for the transformation into GSPCs

**Fig. 6:**
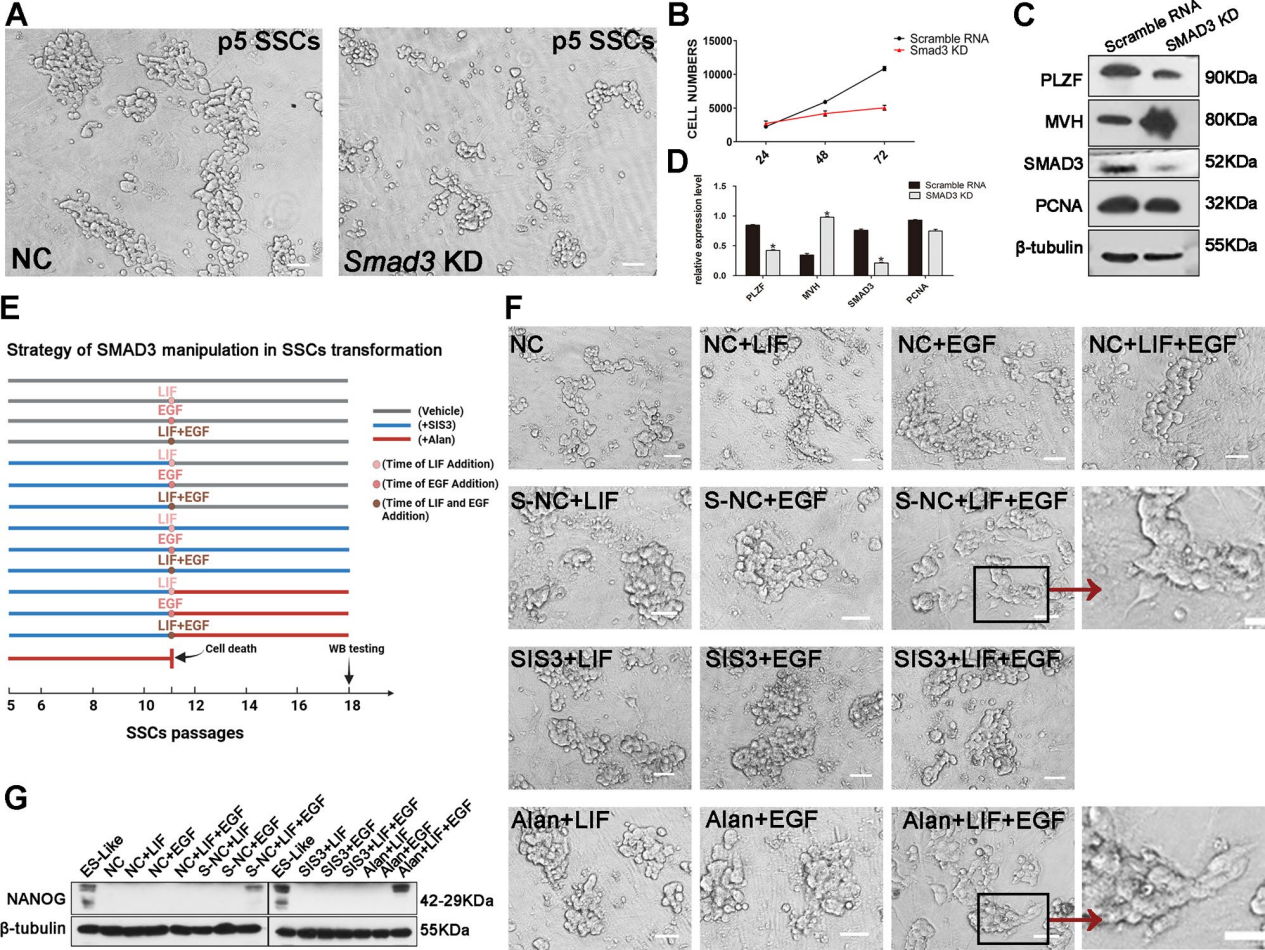
SMAD3 Inhibition and its role in early stage of SSCs pluripotent transformation. (**A**) SSCs morphology following scrambled/*Smad3* siRNA treatment. (**B**) Growth dynamics of SSCs post-treatment. (**C**) Western blot analysis for PLZF, MVH, SMAD3 and PCNA in SSCs treated with scrambled/Smad3 siRNA. (**D**) Statistical interpretation of expression data (*n* = 3). (**E**) Long-term modulation of SMAD3 and the addition of various growth factors to discern SMAD3’s regulatory role in SSCs transformation. (**F**) Morphological assessment of SSCs in different treatment groups, specifically noting cellular changes in the S-NC + LIF + EGF and Alan + LIF + EGF groups. (**G**) Western blot detection of NANOG expression. Data are expressed as mean ± SD (*p < 0.05; **p < 0.01). Scale bars, 20 μm

To further verify the function of SMAD3 and to mitigate potential off-target effects associated with pharmacological interventions, we employed siRNA-mediated knockdown of *Smad3*. After knockdown of *Smad3* in SSCs of 5 passages, a discernible reduction in the size of SSCs population was noted, but the characteristic grape-like cluster morphology of the cells was preserved (Fig. 6A). Quantitative analyses confirmed decreased proliferation rates and SSCs counts upon *Smad3* silencing (Fig. 6B). Intriguingly, despite transient suppression of the expression of the SSCs marker PLZF, the expression of the germ cell marker MVH was elevated following *Smad3* knockdown (Fig. 6C and D). To further elucidate the role of SMAD3 in the transformation of SSCs, we subjected both control and SMAD3-inhibited cohorts to culture for five passages, with and without the addition of EGF and LIF—factors known to markedly expedite transformation [44]. The control group was stratified into four subgroups: a negative control group without EGF and LIF, a group treated with LIF alone, a group treated with EGF alone, and a group treated with both LIF and EGF. Concurrently, the SIS3-treated cells were segregated into three distinct groups: cessation of inhibition (SIS3-vehicle, abbreviated as S-NC), sustained inhibition (SIS3), and augmented activation (Alan), each receiving LIF, EGF, or their combination (Fig. 6E). Notably, morphological alterations were exclusively observed in cells exposed to the synergistic effects of LIF and EGF within the S-NC and Alan groups. This phenotypic transition was characterized by a departure from the archetypal grape-like string morphology of SSCs to colonies with irregular and flattened peripheries (Fig. 6F) and increased expression of the pluripotency marker NANOG (Fig. 6G). These observations support the hypothesis that SMAD3 inhibition in primary SSCs may selectively influence cell subpopulations, modulating growth kinetics and profoundly increasing SSCs proliferation while simultaneously bolstering cellular homeostasis.

### Evaluation of the role of SMAD3 in SSCs via transcriptomic analysis

**Fig. 7:**
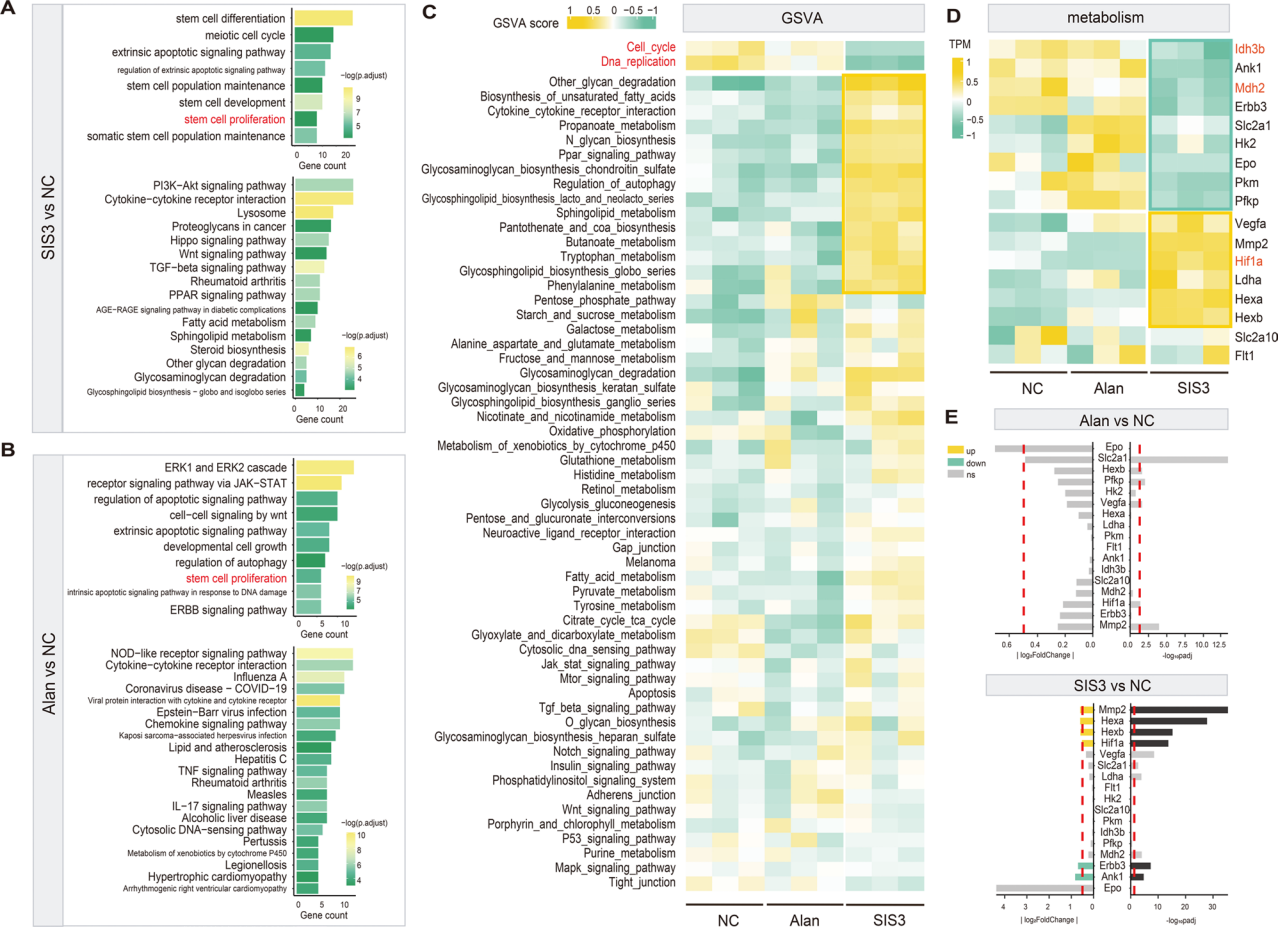
Transcriptomic Landscape of SSCs Modulated by SMAD3 Inhibition or Activation. (**A**) GO (top) and KEGG (bottom) pathway enrichment analyses reveal biological processes in SIS3-treated SSCs relative to controls. (**B**) GO (top) and KEGG (bottom) pathway enrichment analyses comparing Alan-treated SSCs to controls, indicating distinct regulatory cascades. (**C**) GSVA identifies metabolism-related pathways preferentially activated in the SIS3 group. (**D**) Heatmap and (**E**) barplot visualization of gene expression changes linked to glycolysis and the tricarboxylic acid (TCA) cycle in response to SIS3 or Alan treatment

To delineate the role of SMAD3 in the regulation of SSCs, we conducted a comprehensive transcriptomic analysis of SSCs treated with SIS3 and alantolactone. PCA demonstrated robust intragroup consistency and significant intergroup divergence among the SMAD3 inhibition, SMAD3 activation, and control cohorts (Figure S5A). Compared with the control cells, the SIS3-treated cells exhibited 376 upregulated and 295 downregulated genes (Figure S5C and Table S7), while the alantolactone group exhibited 80 upregulated and 157 downregulated genes (Figure S5B and Table S6). Gene Ontology (GO) analysis indicated that the DEGs in the SMAD3-inhibited group were primarily involved in stem cell maintenance, apoptosis signaling, development, and differentiation, suggesting that SMAD3 suppression modulates the expression of genes pivotal for stem cell functionality and fate determination (Fig. 7A top and Table S9). Kyoto Encyclopedia of Genes and Genomes (KEGG) pathway analysis of these DEGs highlighted their enrichment in signaling pathways, including the TGF-β, PI3K-AKT, Hippo, Wnt, and PPAR pathways, indicating a repressive influence of SMAD3 on these signaling cascades (Fig. 7A bottom and Table S10). Furthermore, GO analysis revealed that the DEGs in the SMAD3-activation group were associated with apoptosis, autophagy, and stem cell proliferation (Fig. 7B and Table S8). KEGG analysis revealed enrichment of genes involved in inflammatory and immune responses, linking SMAD3 activity to stem cell fate, proliferation, and survival (Fig. 7B and Table S11). Notably, the changes in the expression of *Hmga2* and *Rbpj*, which are involved in stem cell proliferation, in the Alan and SIS3 groups were consistent with those induced by SMAD3, and protein‒protein interaction (PPI) analysis using the STRING database confirmed the connections among *Rbpj*, *Hmga2*, and *Smad3* (Figure S5D and S5E). Moreover, it has been reported that SMAD3 interacts with RBPJ to activate the expression of the inflammatory factor IL9 [5], suggesting that SMAD3 activation by alantolactone may trigger an inflammatory response to promote cell death. Gene set variation analysis (GSVA) further revealed the downregulation of pathways related to the cell cycle and DNA replication in SMAD3-inhibited cells (Fig. 7C and Table S12), suggesting that SMAD3 may play a role in promoting SSCs proliferation. This is consistent with previous data that SMAD3 regulates cell proliferation for survival during initial stage of SSCs transformation.

However, it’s worth noting that the rapid proliferation is pivotal for SSCs transformation, the observed downregulation of SMAD3, which inhibits SSCs proliferation during the early stages of transformation, is intriguing. The DEGs associated with metabolic pathways revealed the activation of several metabolic processes, including galactose, fructose, mannose, and pyruvate metabolism; glycolysis; gluconeogenesis; and fatty acid metabolism. Considering that galactose, fructose, and mannose metabolism are connected with glycolysis and the pentose phosphate pathway and are critical for the generation of energy and the synthesis of nucleotides and glycoproteins and considering that fatty acid metabolism can provide additional energy through β-oxidation and supply precursors for the synthesis of lipids for membrane biosynthesis during cell growth and division, we concluded that the upregulation of these pathways met the biosynthetic and energetic needs of cells undergoing fate changes. On the other hand, the average expression levels of genes of the pentose phosphate pathway and the TCA cycle, as determined by GSVA, did not markedly change (Fig. 7C), but the expression levels of *Mdh2* and *Idh3b* (critical enzymes in the TCA cycle), with IDH3B acting as the rate-limiting enzyme of the TCA cycle [21], were markedly downregulated (Fig. 7D and E), suggesting that the efficiency of the TCA cycle was inhibited in the transforming cells. Moreover, the suppressed expression of genes associated with the cell cycle and DNA replication further confirmed that the cells were in a transition state in which growth was arrested, probably due to metabolic reprogramming.

To understand the underlying mechanism, we focused on HIF-1α, a pivotal regulator of glycolysis and the TCA cycle, which is known to bind the MH2 domain of phosphorylated SMAD3 to influence the metabolic switch through TGF-β signaling [10]. We observed marked upregulation of HIF-1α and related genes, such as Vascular Endothelial Growth Factor A (*Vegfa*) and Lactate Dehydrogenase A (*Ldha*), as well as downregulation of Malate Dehydrogenase 2 (Mdh2) and Isocitrate Dehydrogenase 3 (NAD(+)) beta (*Idh3b*), in the SMAD3-inhibited group (Fig. 7D and E). This pattern suggests activation of glycolysis and suppression of the TCA cycle, potentially mediated by HIF-1α, aligning with our hypothesis that aerobic glycolysis is favored during SSCs transformation. The inhibition of SMAD3 in primary SSCs may thus prompt metabolic reprogramming, which facilitates energy production and activates pathways conducive to rapid cell proliferation.

### Inhibition of SMAD3 promoted aerobic Glycolysis in SSCs and accelerated SSCs transformation

**Fig. 8:**
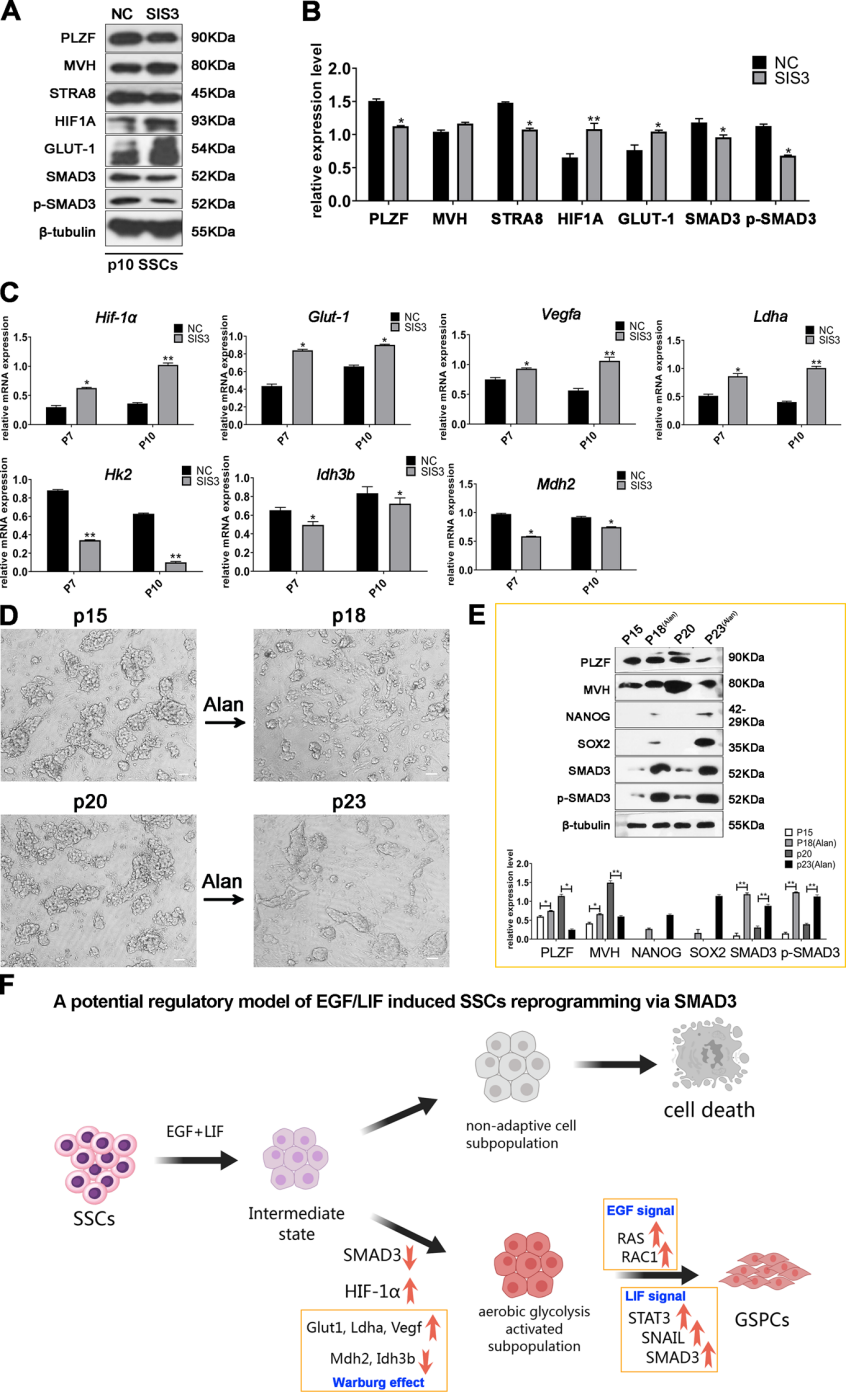
Metabolic reprogramming and pluripotent transition of SSCs influenced by SMAD3 modulation. (**A**) Western blot analysis quantifying the expression of PLZF, MVH, STRA8, HIF1A, GLUT-1, SMAD3, and phosphorylated SMAD3 (p-SMAD3) in SSCs treated with SIS3 (10 µM). (**B**) Statistical assessment of protein expression levels in Figure A (*n* = 3). (**C**) RT-PCR quantification of relative mRNA levels of *Hif-1α*, *Glut-1*, *Vegfa*, *Ldha*, *Hk2*, *Idh3b*, and *Mdh2* in NC versus SIS3 SSCs, with statistical evaluation. (**D**) Morphological examination of SSCs at passages 15 and 20 following three passages of Alan treatment. (**E**) Western blot detection of PLZF, MVH, NANOG, SOX2, SMAD3, and p-SMAD3 across various passages of SSCs with and without Alan treatment, and with statistical analysis (*n* = 3). (**F**) The model for the potential role of SMAD3 in EGF- and LIF-mediated SSCs transformation is exhibited. Results are presented as mean ± SD, with significance denoted by *p < 0.05 and **p < 0.01. Scale bar = 20 μm

To elucidate the regulatory role of SMAD3 in the metabolic reprogramming of SSCs, we examined the effects of SMAD3 modulation on glucose metabolism in p6 SSCs. The inhibition of SMAD3 activity resulted in a transient decrease in the number of SSCs within the first two passages after SIS3 treatment. However, subsequent resurgence and proliferation beyond the baseline rates were observed at p10, indicating selection for a subpopulation with enhanced adaptability (Fig. 5B). Conversely, SSCs subjected to alantolactone treatment exhibited cellular rupture and failed to survive for five more passages (Fig. 5B). Protein expression analysis of p10 SSCs revealed marked upregulation of HIF-1 A and GLUT1, key regulators of aerobic glycolysis, upon SMAD3 inhibition (Fig. 8A and B). RT‒PCR corroborated these findings, showing increased transcription of genes that are known to facilitate glycolysis, including *Hif-1α*, *Glut1*, *Ldha*, and *Vegfa* (Fig. 8C) [14]. Conversely, downregulation of *Mdh2* and *Idh3b*, which are critical enzymes in the TCA cycle, was observed (Fig. 8C), consistent with the transcriptomic results. These changes suggest a metabolic shift in SSCs from oxidative phosphorylation to glycolysis under SMAD3 inhibition. Furthermore, transformation assays revealed the emergence of GSPCs among SSCs exposed to sequential treatment with SIS3 followed by alantolactone, a pattern not detected in control groups undergoing continuous SIS3 treatment (Fig. 6F). This finding suggested that transient inhibition of SMAD3 followed by SMAD3 reactivation may enhance SSCs transformation. We postulate that the previously reported downregulation of SMAD3 during the initial stages of SSCs reprogramming serves to initiate aerobic glycolysis [22]. To test the hypothesis that SMAD3 activation is crucial during the mid-to-late stages of SSCs transformation, particularly after adaptation to aerobic glycolysis caused by SMAD3 inhibition, p15 and p20 SSCs—typically devoid of NANOG expression at the protein level and prone to transformation within further cultures for 10 and 5 passages, respectively—were cultured in medium supplemented with SIS3 or alantolactone. Remarkably, SSCs treated with alantolactone underwent accelerated transformation within just three passages, with the transformation rate at p20 significantly outpacing that at p15 (Fig. 8D and E). This finding underscores the necessity of SMAD3 activation for the induction of transformation in SSCs subjected to prolonged culture.

Integrating our current observations with the conclusions of previous investigations, we propose a model for the potential role of SMAD3 in EGF- and LIF-mediated SSC transformation (Fig. 8F). Initially, downregulation of SMAD3 coincides with HIF-1 A activation, enabling a metabolic switch to aerobic glycolysis. As SSCs adapt to this altered energy landscape, LIF signaling prompts SMAD3 activation, which, in concert with RAS and RAC1 signaling elicited by EGF, promotes pluripotency. However, the intricate details of this process warrant further exploration, as discussed in the Discussion section.

## Discussion

The spontaneous reprogramming of SSCs to a pluripotent state is a fascinating and significant phenomenon uniquely observed in germ line cells. This transformation process is particularly noteworthy as it occurs without the introduction of oncogenes, suggesting potential for safe clinical applications or animal cloning. This phenomenon was first observed two decades ago, occurring in an extremely low efficiency (1 in 1.5 × 10^7^ testicular cells) and over a time span of several months. In our efforts to optimize the growth conditions for SSCs, we occasionally observed that SSCs cultured in medium containing GDNF, bFGF, and 1% FBS on fresh MEF feeders could reprogram within as few as 5 passages with the addition of 10 ng/mL LIF and 20 ng/mL EGF. This rapid dedifferentiation was accompanied by changes not only in cell morphology but also in metabolism-associated signaling pathways, such as the PI3K/AKT/mTOR pathway [44]. This pathway coordinates the uptake and utilization of nutrients, such as glucose and glutamine, and plays a pivotal role in cellular metabolism, including glycolysis and the TCA cycle. It is indispensable for the regulation of aerobic glycolysis, particularly in cells with specific mutations, to support enhanced growth and proliferation [25, 48]. However, the connection between these signaling pathways and metabolic reprogramming in SSCs has not yet been fully elucidated. In this study, we revealed that SMAD3 cooperates with HIF-1 A to regulate the transition from the TCA cycle to aerobic glycolysis during SSCs transformation. We observed a significant increase in glycolytic activity during the early stages of transformation, while the critical enzymes of the TCA cycle, *Mdh2* and *Idh3b* (the rate-limiting enzyme of the TCA cycle*)*, were suppressed. Thus, the TCA cycle was inhibited to divert metabolic flux toward glycolysis and the pentose phosphate pathway. Consistent with these findings, increased levels of 5-phosphoribosyl 1-pyrophosphate, 7-phosphoheptulonate, and 4-phosphoerythronate confirmed the significant activation of the pentose phosphate pathway by SMAD3 inhibition. Considering glycolysis positively impacts SSCs self-renewal, while oxidative phosphorylation gradually intensifies during differentiation [3, 9], the enhanced glycolytic activity during dedifferentiation appears to mirror the Warburg effect observed in reprogramming, possibly serving as a mechanism to facilitate rapid ATP generation and biosynthetic precursor production for rapidly proliferating GSPCs [19].

Notably, there are some differences between SSCs spontaneous transformation and tumor formation in metabolic reprogramming. Observations from Otto Warburg revealed a decreased respiration along with an enhanced lactate production in cancer cells, rates that correlate with increased cell proliferation [27]. However, lactate level during SSCs transformation do not change remarkably. The PI3K/AKT/mTOR and Wnt signaling pathways can regulate the balance between glycolysis and the TCA cycle, ensuring that cells meet their energy requirements while accumulating the building blocks (amino acids, nucleotides, and lipids) needed for rapid proliferation [40]. This explains why the observed increase in glycolytic activity did not lead to lactate accumulation. The enhancement of gluconeogenesis likely serves as a protective mechanism, reducing lactate-induced cellular damage while maintaining elevated levels of glycolysis and the pentose phosphate pathway. This, in turn, provides the precursors required for the synthesis of nucleotides, amino acids, and lipids. Therefore, we posit that the SMAD3-mediated metabolic reprogramming in SSCs, in contrast to tumor cells, likely represents a more controlled regulatory process that mitigates cellular damage. The Warburg effect in tumor cells involves distinct metabolic pathways, leading to significant divergences in cellular fate and characteristics. Notably, lactate concentration is closely associated with cellular fate determination. Evidence suggests that elevated lactate levels can influence gene expression by modifying m6A RNA modifications and can also induce cellular damage, such as ferroptosis [45]. Consequently, the relative stability of lactate levels during the spontaneous reprogramming of SSCs may be correlated with maintaining cellular homeostasis, distinguishing them from neoplastic cells in terms of metabolic regulation and cellular stability.

Another distinguishing feature lies in the spontaneous reprogramming process of SSCs, characterized by dynamic changes in SMAD3 expression. This process can be bifurcated into two distinct phases: an initial downregulation that activates metabolic reprogramming, followed by a subsequent upregulation that promotes cellular proliferation. This unique pattern of SMAD3 expression and its functional consequences represent a significant departure from the mechanisms observed in tumor cells [6]. During the initial phases of SSCs transformation, the cellular focus shifts from energy production to the synthesis of essential biomolecules. Despite the absence of a significant increase in overall TCA cycle activity, the increased succinate level sufficiently fulfills the basal energy demands of the cells. Following transformation, the cellular metabolic pattern underwent another significant change, with ATP levels recovering to approximately twice those of SSCs, and an increase in TCA cycle activity. This indicates that upon completion of the reprogramming process, cells efficiently meet both the material and energy requirements for rapid proliferation, which is crucial for maintaining cellular energy homeostasis. Overall, the state of cellular energy metabolism, as indicated by the activities of the TCA cycle, glycolysis and the pentose phosphate pathway, can serve as a critical indicator of cellular fate, reflecting the transition of SSCs from a relatively quiescent state with a low degree of stemness to a highly active state with a high degree of stemness (pluripotency).

High glutamine metabolism is crucial for pluripotent stem cells by preserving OCT4 through maintaining endogenous antioxidant glutathione (GSH), which reduces oxidation of OCT4 cysteine residues essential for DNA binding and stability [26]. Here, we were particularly intrigued by the specific alterations in the levels of glutamine and glutamate, which markedly increased in both the *In state* cells and GSPCs (Table S3, S4 and S5), implying a molecular connection of metabolic change and pluripotency acquirement in SSCs reprogramming. Concurrently, we noted elevated levels of various metabolites, such as tyrosine, arginine, lysine, threonine, and specific nucleotides, underscoring the vital role of glutamine even when glucose levels are stable. However, when we tested whether high doses of Gln affect SSCs transformation, we unexpectedly found that both high doses of Gln (4 mM) and the removal of Gln hinder the growth of SSCs and may even have substantial impacts (data not shown). Glutamine performs a variety of essential functions, from participating in the synthesis of amino acids, nucleotides, and lipids to aiding in protein synthesis, transport, and resistance to ROS damage [38]. Glutamine appears to counterbalance the increased biosynthetic and energetic demands associated with the dedifferentiation process, thereby sustaining the growth and metabolic activity of GSPCs. In cancer cells, the preferential utilization of glutamine becomes critically important for maintaining biosynthetic and energetic stability, especially when glucose availability is limited [1]. However, under normal SSCs culture conditions, a high level of glutamine might affect metabolic patterns and substantially decrease endogenous ROS levels, essential for SSCs maintenance and self-renewal [31], leading to arrested cell growth. Therefore, these observations indicate that increasing the concentration of glucose or glutamine does not enhance the spontaneous reprogramming efficiency of SSCs; instead, it impacts cell survival. This suggests that these metabolites may accumulate to play essential roles in promoting further reprogramming but are not critical factors for triggering reprogramming.

Moreover, the metabolic characteristics of germline stem cells may differ significantly from those of other stem cells. For instance, while embryonic stem cells and bone marrow stem cells prefer glycolysis for energy production, PGCs at embryonic day 13.5 tend to rely on oxidative phosphorylation [8]. This metabolic preference is gradually established during PGC differentiation and is maintained until the formation of SSCs, indicating a predilection for oxidative phosphorylation in germline stem cells. However, during SSCs spontaneous reprogramming, a critical event known as “metabolic reprogramming” occurs. This phenomenon is characterized by a metabolic shift from oxidative phosphorylation to glycolysis, suggesting that alterations in cellular metabolism are intricately linked to changes in cell fate. This metabolic transition coincides with the loss of germline-specific characteristics and the acquisition of pluripotent stem cell properties, underscoring the fundamental role of metabolic plasticity in cellular reprogramming and fate determination.

The connection of HIF-1 A and SMAD3 in our study also draws attention to the mechanism of SMAD3 in the regulation of metabolic reprogramming. Studies have demonstrated a positive correlation between the expression patterns of SMAD3 and HIF-1 A [10]. However, upon the inhibition of SMAD3 activity using specific inhibitors, we observed the activation of HIF-1 A expression. We postulate that this may be attributable to a feedback regulatory mechanism: the suppression of SMAD3 activity triggers feedback regulation through direct or indirect mechanisms, leading to an increase in HIF-1 A expression. Alternatively, this could be due to a compensatory effect. When SMAD3 signaling is inhibited, cells might activate an alternative pathway to upregulate HIF-1 A expression. Given the concurrent activation of the PI3K-AKT-mTOR signaling pathway during the intermediate state of transformation, and studies reported that PI3K-AKT-mTOR signaling pathway could induce expression of Hif1α [15], it is plausible that this upregulation of HIF-1 A is associated with the PI3K-AKT-mTOR axis. Nevertheless, this hypothesis requires further experimental validation.

In summary, the inhibition of SMAD3 in SSCs during the early stage of transformation appears to induce a metabolic shift toward aerobic glycolysis, thereby facilitating the provision of sufficient energy to support rapid cellular proliferation. As the subpopulation acclimatizes to the new growth and metabolic patterns, activation of SMAD3 promotes transformation. However, activation of SMAD3 in primary SSCs may trigger increased expression of inflammatory factors that subsequently impact cell maintenance, suggesting that the shift to aerobic glycolysis is a critical step for further transformation.

## Conclusion

This study elucidates the critical role of SMAD3 in regulating SSCs fate, highlighting the complex interplay between molecular signaling and energy metabolism. Our findings suggest that metabolic reprogramming, mediated by SMAD3, is crucial for the transformation of germline stem cells into a pluripotent state. The potential applications of these findings are far-reaching, from novel treatments for male infertility and testicular cancer to enhancing the efficiency of animal cloning. However, caution must be exercised due to potential unforeseen consequences of manipulating fundamental cellular processes. While some questions remain unanswered, such as the long-term effects of SMAD3 manipulation on genomic stability or cell metabolism, this research opens new avenues for innovative therapeutic approaches in reproductive health and stem cell biology. These insights underscore the importance of interdisciplinary research in unraveling complex biological processes and pave the way for future breakthroughs in regenerative medicine and reproductive science.

## Materials and methods

### Animal procurement and ethics

The study utilized ICR mice obtained from Nanjing Medical University (Nanjing, China), and were housed in the Specific Pathogen-Free (SPF) Animal Facility at Nanjing Agricultural University under standard laboratory conditions. Euthanasia of mice was performed in accordance with guidelines of Nanjing Agricultural university and ethical committee. Mice were euthanized using CO₂ inhalation in a gradually filled chamber, followed by cervical dislocation to ensure death. All procedures were conducted to minimize pain and distress.

### Spermatogonial stem cell isolation and culture

Spermatogonial stem cells (SSCs) were isolated from the testes of male mice aged 5–6 days as previously described [36]. Briefly, after the excision of the tunica albuginea, the testicular tissue was minced and enzymatically dissociated with collagenase IV (Amresco, WDC, USA, #17104-019) at 37 °C for 15 min. The tissue was then washed with D-Hanks solution, and seminiferous tubule fragments were further digested with 0.05% trypsin (Amresco, WDC, USA, #27250-018) at 37 °C for 5 min. The resultant cell suspension, after centrifugation and removal of enzymatic solutions, contained a mixture of preliminary SSCs and other testicular cells. SSCs were then enriched using two rounds of differential plating. The purified SSCs were cultured on a feeder layer of mitomycin C (Sigma-Aldrich, St. Louis, MO, USA, #M5353)-treated mouse embryonic fibroblasts (MEFs), prepared as previously described. Cultivation occurred in 24-well plates using Shinohara’s Iscove’s Modified Dulbecco’s Medium (IMDM, Gibco, Grand Island, NY, USA, #12200-069) supplemented with fetal bovine serum (FBS, Gibco, Grand Island, NY, USA, #16000-044), with the cultures maintaining stability over 40 passages in vitro.

### RNA extraction, reverse transcription, and semi-quantitative PCR

Total RNA from SSCs was extracted using TRNzol reagent (Tiangen, Beijing, China, #DP424) per the manufacturer’s instructions. cDNA synthesis was performed using the GoScript™ Reverse Transcription System (Promega, Madison, WI, USA, #A5001). Semi-quantitative polymerase chain reaction (PCR) was conducted to assess gene expression, employing Premix Ex Taq (Takara, Otsu, Shiga, Japan, #RR390A) and an Eppendorf PCR system (Hamburg, Germany). GAPDH served as an internal reference. Primer sequences are detailed in Table S1. PCR products were resolved on 1.5% agarose gels stained with ethidium bromide, visualized with a Tanon scanner (Shanghai, China), and analyzed using ImageJ software (National Institutes of Health, Bethesda, MD, USA).

### Immunofluorescence assay and western blot

Immunofluorescence staining of SSCs was carried out as previously delineated [43]. Cells were fixed with Carnoy’s solution for 20 min at -20 °C, washed with PBS, and blocked with 10% goat serum for 30 min at room temperature. Incubation with primary antibodies proceeded overnight at 4 °C, followed by secondary antibody application for 1 h after PBS washes. Nuclei were stained with Hoechst 33,342 (CST, Danvers, MA, USA, #4082), and cells were examined under a fluorescence microscope (OLYMPUS, Tokyo, Japan).

The Western blotting procedure was conducted as previously described [43], with the following steps briefly outlined: protein lysates were resolved by SDS-PAGE and subsequently transferred onto PVDF membranes (Merck Millipore, Billerica, MA, USA). Membranes were blocked with 5% milk for 1 h at room temperature before the addition of the primary antibodies, and were incubated at 4 °C overnight. Following incubation, membranes were washed twice with TBST. Detection of the primary antibodies was performed using peroxidase-conjugated goat anti-rabbit IgG or goat anti-mouse IgG secondary antibodies. Immunoreactive bands were visualized using enhanced chemiluminescence (ECL, Biosharp, Hefei, China, #BL520A) and exposed to film. The intensity of the bands was quantified utilizing ImageJ software.

Antibodies used in this study are listed in Table S2.

All full-length blots/gels are presented in Supplementary Figure S6.

### siRNA-mediated knockdown

SiRNAs targeting smad3 were synthesized by Genepharma (Shanghai, China) and transfected into SSCs using Lipofectamine 3000 (Life Technologies, Carlsbad, CA, USA, #L3000-015) as per the manufacturer’s protocol. Cells were collected for analysis. The information of siRNA is listed below. Smad3 siRNA: 5’-AUACGAUAGAUCAGUGGGAtt-3’, 5’-UCCCACUGAUCUAUCGUAUtt-3’.

### Targeted metabolomics analysis

Targeted metabolomics analysis was performed to quantify specific metabolites associated with aerobic glycolysis, TCA cycle intermediates, and epigenetic reprogramming pathways. The targeted approach was selected to validate hypotheses derived from our prior transcriptomic studies and to ensure absolute quantification of predefined metabolic markers.

Analytical-grade reagents, including acetonitrile (ACN) and methanol (MeOH), were sourced from Merck (Darmstadt, Germany), alongside ultrapure water obtained from a Milli-Q system (Millipore, Bradford, USA). Certified analytical standards for target metabolites were procured from Sigma-Aldrich (St. Louis, MO, USA) and other certified providers, with formic acid also supplied by Sigma-Aldrich. These standards were prepared in methanol to a primary concentration of 1 mg/mL, supplemented with isotope-labeled internal standards to correct for matrix effects and recovery variations, and stored at -20 °C. Working solutions were freshly prepared by appropriate dilution in methanol prior to analysis.

Sample preparation involved thawing on ice, followed by homogenization with 500 µL of 80% methanol/water, pre-cooled to -20 °C, and vigorous vortexing. This procedure was repeated thrice after snap-freezing in liquid nitrogen and thawing. Centrifugation at 12,000 × g for 10 min at 4 °C allowed for the collection of the supernatant, which was subsequently incubated at -20 °C for 30 min and re-centrifuged under identical conditions. To enhance specificity for target metabolites, the clarified supernatant was processed through a protein precipitation plate (Agilent, USA) optimized for polar and semi-polar small molecules, in preparation for LC-MS analysis.

The metabolite extracts were analyzed on an integrated LC-ESI-MS/MS system comprising a UPLC (ExionLC AD, SCIEX) coupled to a QTRAP^®^ 6500 + mass spectrometer (SCIEX). Chromatographic separation was achieved using an ACQUITY UPLC BEH Amide column (2.1 × 100 mm, 1.7 μm) at 40 °C, employing a binary solvent system of water with 10 mM ammonium acetate and 0.3% ammonium hydroxide (solvent A) and 90% ACN (solvent B). The gradient commenced at 95% B, reduced to 70% over 8 min, then to 50% B for 2 min, and subsequently returned to 95% B by 15 min, with a total run time of 20 min to ensure complete elution of target metabolites. The flow rate was set at 0.4 mL/min and an injection volume of 2 µL.

Mass spectrometric detection was facilitated by an AB 6500 + QTRAP^®^ system, featuring an ESI Turbo Ion-Spray interface and operated under both positive and negative ion modes. The system was managed using Analyst 1.6 software (AB Sciex). The ESI source parameters included a turbo spray ion source at 550 °C, ion spray voltages of 5500 V for positive and − 4500 V for negative modes, and a curtain gas pressure of 35.0 psi. Multiple reaction monitoring (MRM) transitions were optimized for each analyte, with the selection of specific MRM transitions based on the elution profiles of plant hormones.

Metabolite identification and quantification were performed by MetWare (http://www.metware.cn/) using the AB Sciex QTRAP 6500 LC-MS/MS platform, ensuring high specificity and sensitivity in the detection of the metabolic profiles. Absolute quantification was achieved by interpolating peak areas against standard curves (R² >0.99), with recovery rates between 85% and 115% for all target metabolites. Data were normalized to total protein content measured by BCA assay.

### Bioinformation analysis

RNA-seq data were quality-controlled using FastQC (v0.11.9) and low-quality reads and adapters were removed using Trim Galore (0.6.10). The clean data were then aligned to the mm39 reference genome using HISAT2 (2.1.0), and reads were counted using featureCounts (2.0.3). Expression levels were represented by TPM (Transcripts Per Kilobase per Million mapped reads), and differentially expressed genes (DEGs) were identified using DEseq2 (1.38.3) with the criteria|log2FoldChange|≥1 and adjusted p-value < 0.05. GO enrichment analysis of DEGs was performed using clusterProfiler (4.6.1). Gene set variation analysis (GSVA) was conducted using GSVA (1.46.0). The alignment of reads were visualized using the IGV software. By utilizing the STRING database (https://www.string-db.org/), protein-protein interaction (PPI) analysis was conducted. Subsequently, the PPI network diagram was generated using Cytoscape software (v3.10.0).

Metabolomic analysis was performed using a liquid chromatography-tandem mass spectrometry (LC-MS/MS) platform and a custom-built database, detecting a total of 42 metabolites. Sample data were clustered and visualized using ComplexHeatmap (2.14.0). KEGG enrichment analysis of metabolites specifically upregulated in GSPCs was conducted using the online tool MetaboAnalyst 6.0 (https://www.metaboanalyst.ca/MetaboAnalyst/), with pathways identified as significantly enriched if FDR < 0.05.

RRBS-seq data were quality-controlled using FastQC (v0.11.9) and low-quality data were filtered using Trim Galore (0.6.10). Subsequently, Bismark (v0.23.1) was used for alignment to the mm39 reference genome, and methylation sites were extracted using the bismark_methylation_extractor function. The methylation status of promoter regions was visualized using the IGV software. Differential methylation analysis and methylation annotation were performed using MethylKit (1.24.0).

The original data of RNA-seq were from https://www.ebi.ac.uk/fg/annotare/login/#list:all, and E-MTAB-12,342 is the code to review the original data [44]. The original data of Metabolomic assay has been uploaded to www.ebi.ac.uk/metabolights/MTBLS12698, and MTBLS12698 is the code to review the original data. The RRBS-seq data were deposited in the National Center for Biotechnology Information (NCBI) Sequence Read Archive (SRA) repository with accession numbers PRJNA1115908.

### Statistical analysis

Quantitative assessment of cell populations was executed via random selection of fields within immunofluorescence preparations. Enumeration of all spermatogonia was conducted under a 200× magnification field of a microscope, followed by statistical evaluation. For SSCs counting, SSCs were tallied utilizing a hemacytometer after each passage. Statistical significance was ascertained employing Student’s t-test. Data synthesis and graphical representations were performed with the aid of Excel and GraphPad Prism version 8 (GraphPad, San Diego, CA). Results are depicted as mean ± standard deviation (SD), with statistical significance thresholds set at *p < 0.05 and **p < 0.01.

The work has been reported in line with the ARRIVE guidelines 2.0.

## Electronic supplementary material

Below is the link to the electronic supplementary material.

Supplementary Material 1

Supplementary Material 2

Supplementary Material 3

Supplementary Material 4

Supplementary Material 5

Supplementary Material 6

Supplementary Material 7

Supplementary Material 8

## Data Availability

The data that supports the findings of this study are available in the method part and supplemental materials. The links and codes of the raw data of RNA-seq, RRBS-seq and metabolomics are listed in the Method section.

## Abbreviations

DEGs: Differential expressed genes
EGF: Epidermal growth factor
ESC: Embryonic stem cells
FBS: Fetal bovine serum
GDNF: Glial cell line-derived neurotrophic factor
GSPCs: Germline stem cells derived pluripotent cells
GSVA: Gene Set Variation Analysis
IMDM: Iscove’s modified Dulbecco’s medium
iPSCs: Induced pluripotent stem cells
LIF: Leukemia inhibitory factor
MEF: Mouse embryonic fibroblast
PCA: Principal component analysis
SSCs: Spermatogonial stem cells
TCA: Tricarboxylic acid
TGF-β: Transforming Growth Factor Beta
