# Supplementary figures and images for: SMAD3 and HIF-1α orchestrate metabolic transition to aerobic glycolysis as a critical prerequisite for spontaneous reprogramming of spermatogonial stem cells
Source: Stem Cell Res Ther. 2025 Jul 28;16:411. doi: 10.1186/s13287-025-04541-w (PMC12305914; doi:10.1186/s13287-025-04541-w)

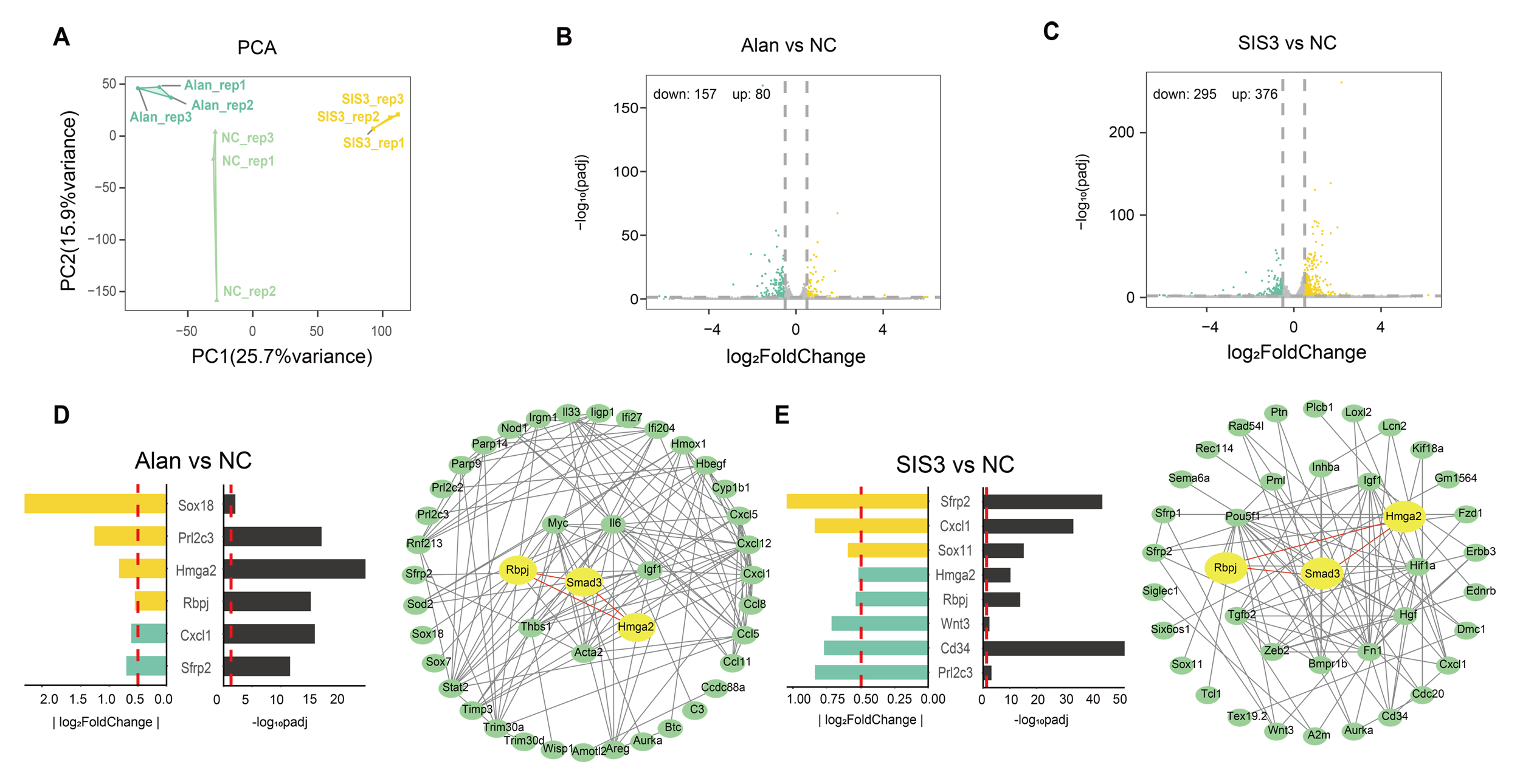

Supplement: Supplementary file 1 — Supplementary Material 1 [file 13287_2025_4541_MOESM1_ESM.tif]

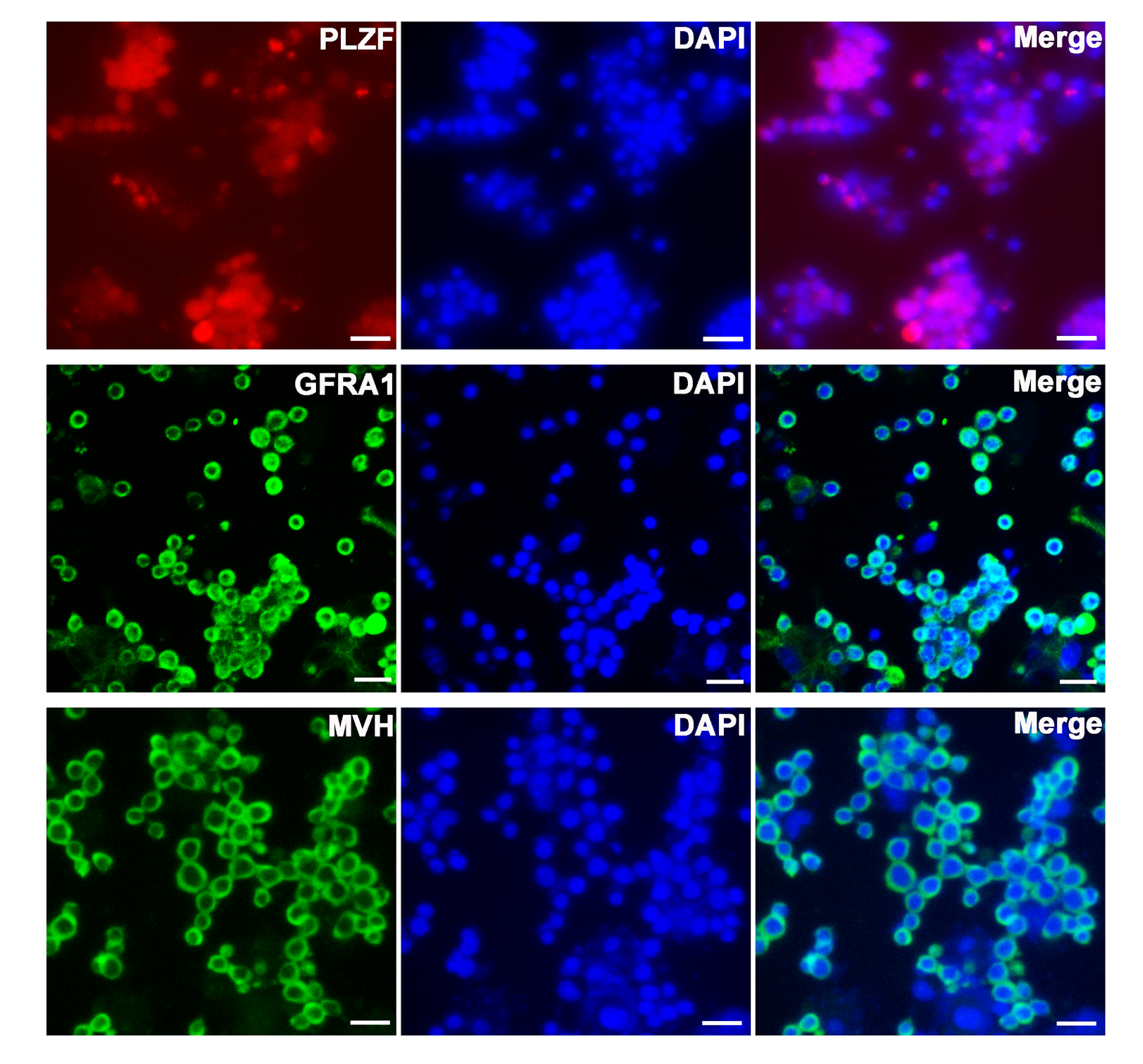

Supplement: Supplementary file 2 — Supplementary Material 2 [file 13287_2025_4541_MOESM2_ESM.tif]

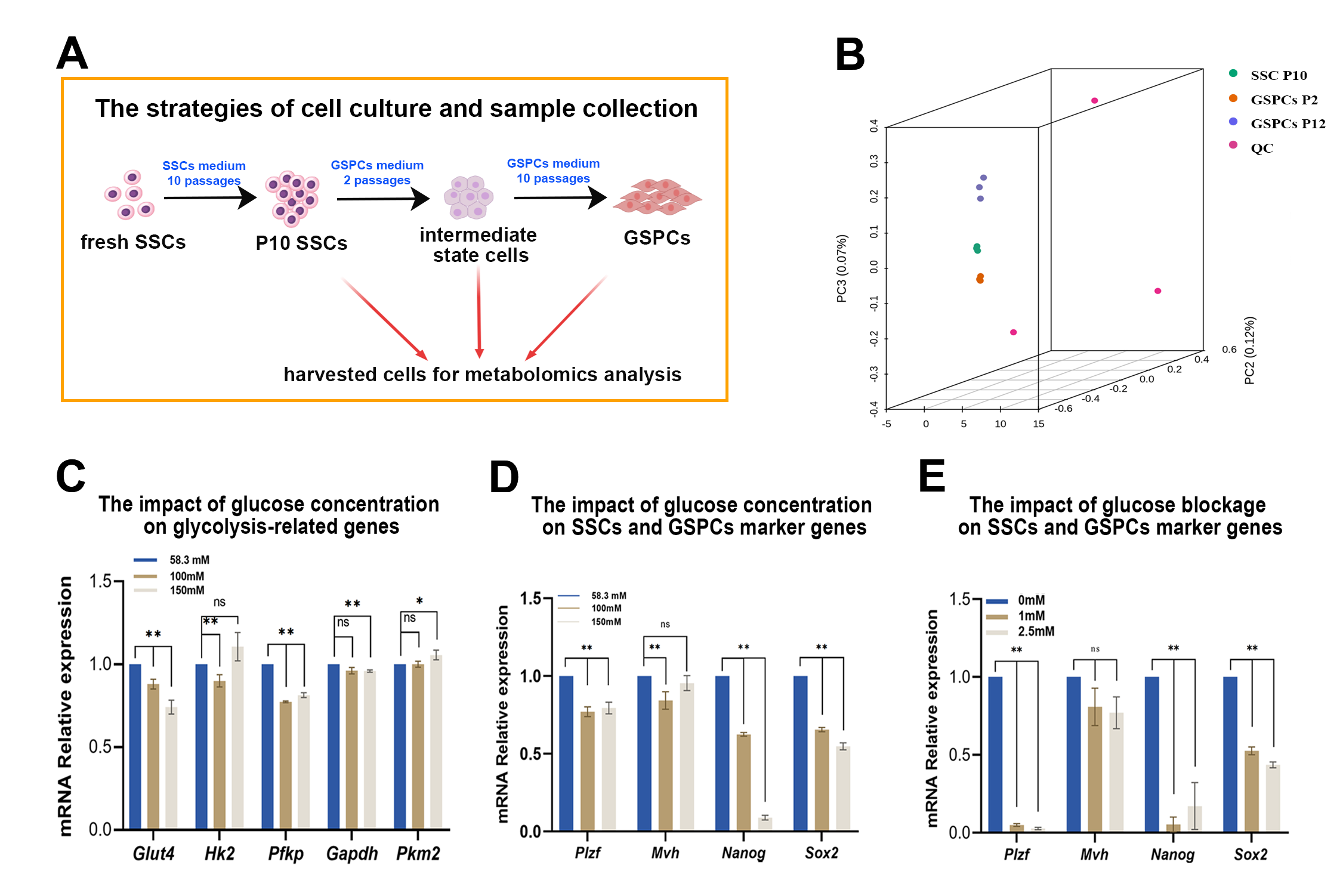

Supplement: Supplementary file 3 — Supplementary Material 3 [file 13287_2025_4541_MOESM3_ESM.tif]

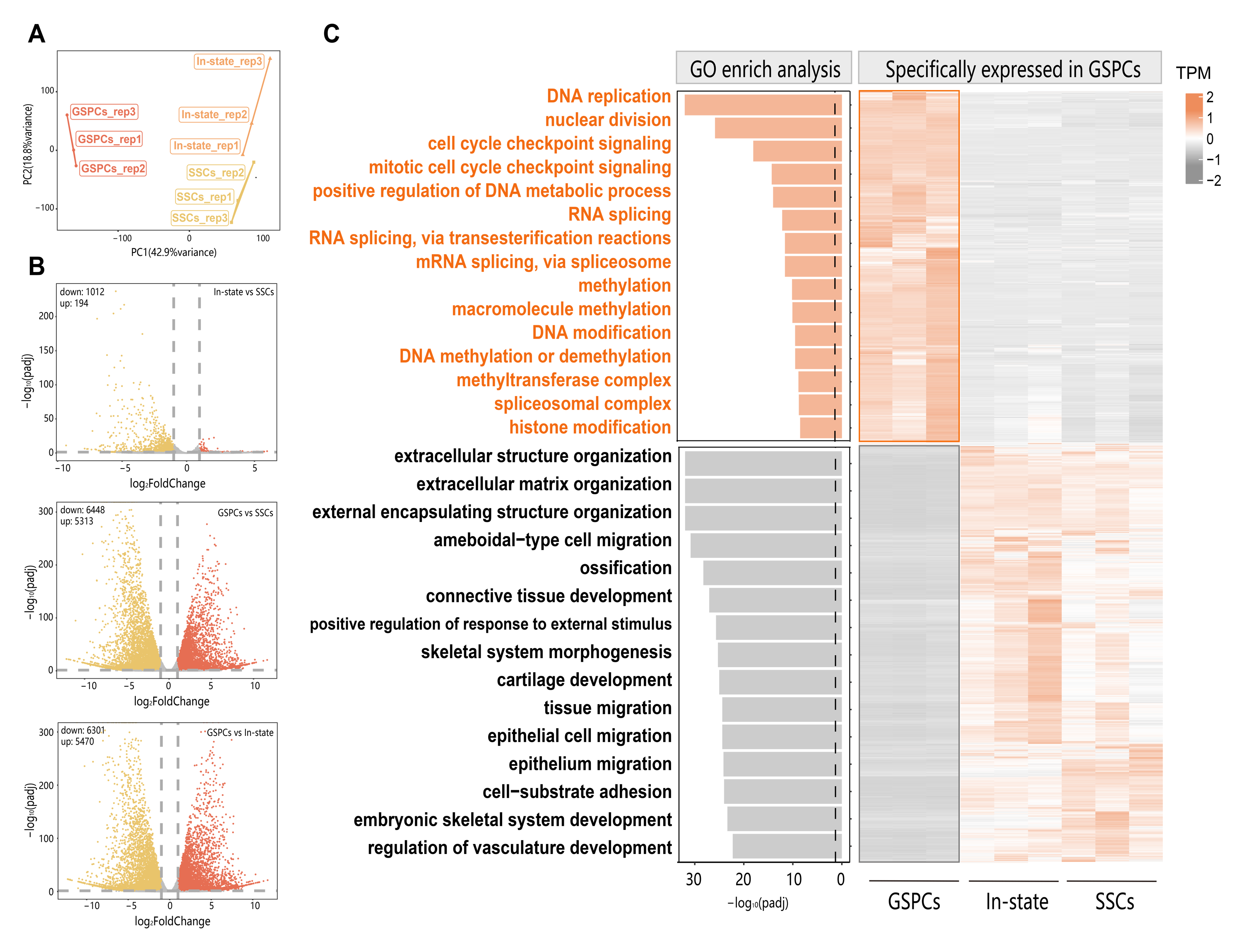

Supplement: Supplementary file 4 — Supplementary Material 4 [file 13287_2025_4541_MOESM4_ESM.tif]

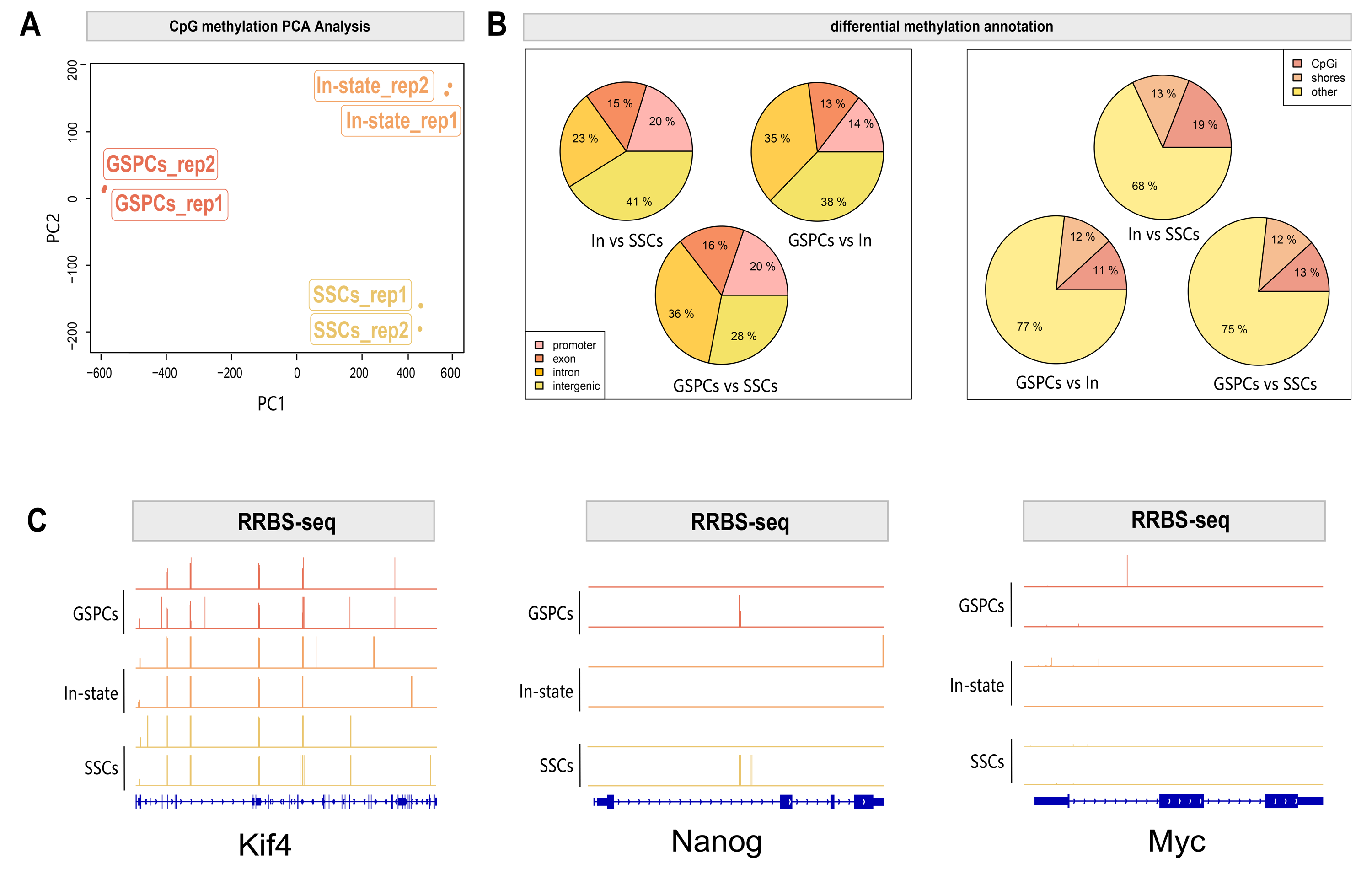

Supplement: Supplementary file 5 — Supplementary Material 5 [file 13287_2025_4541_MOESM5_ESM.tif]

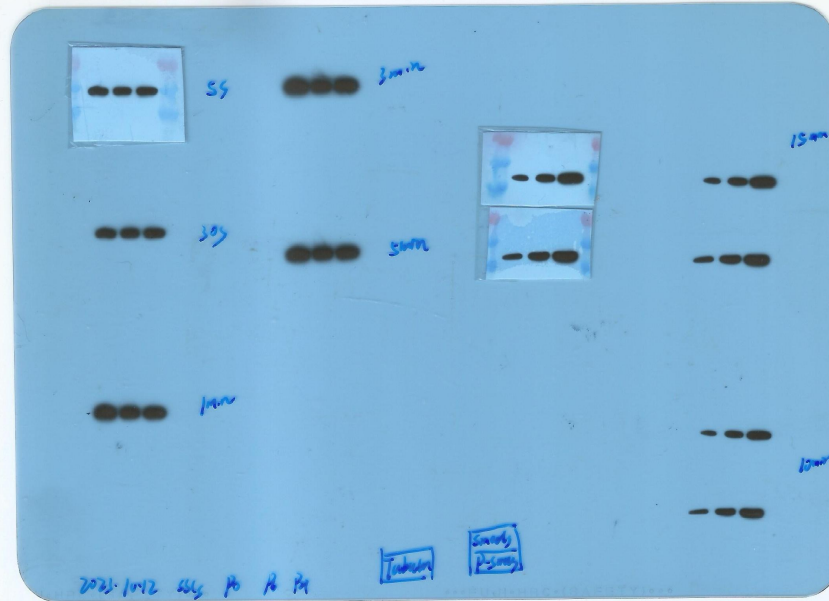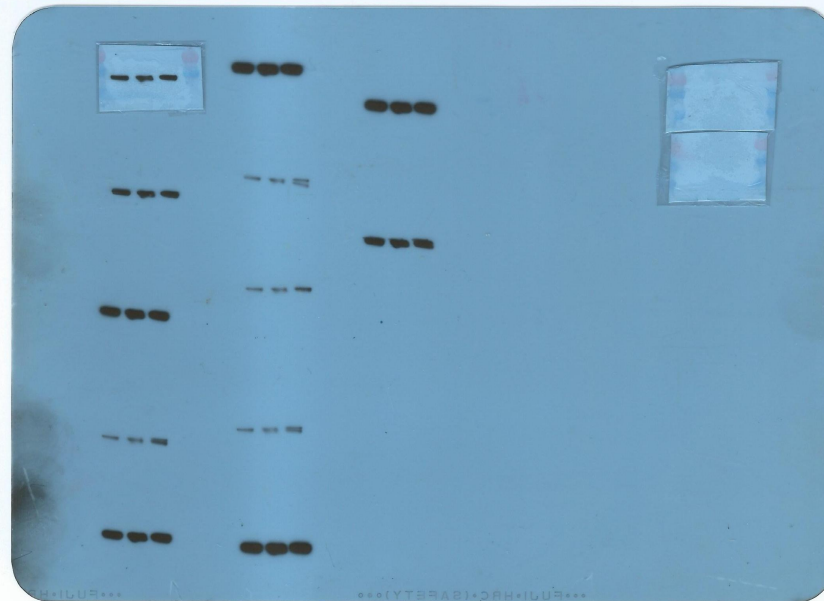

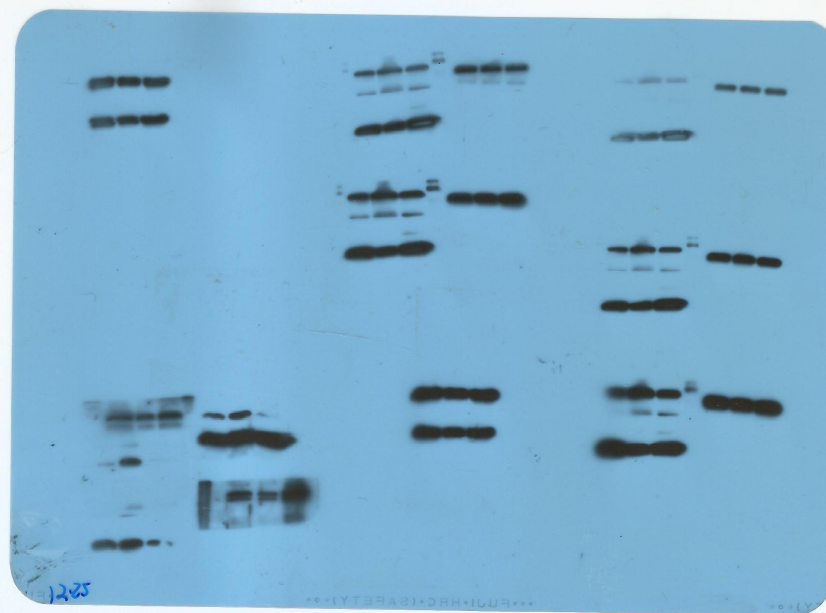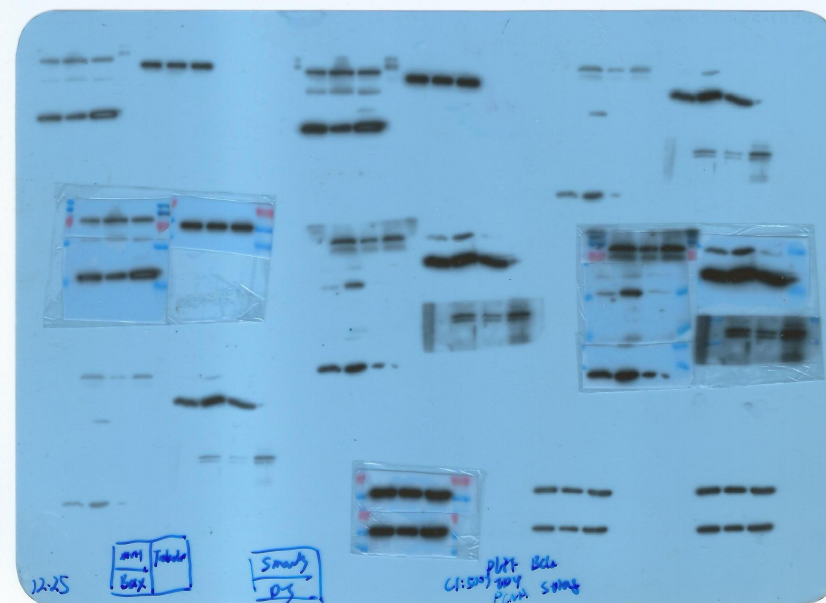



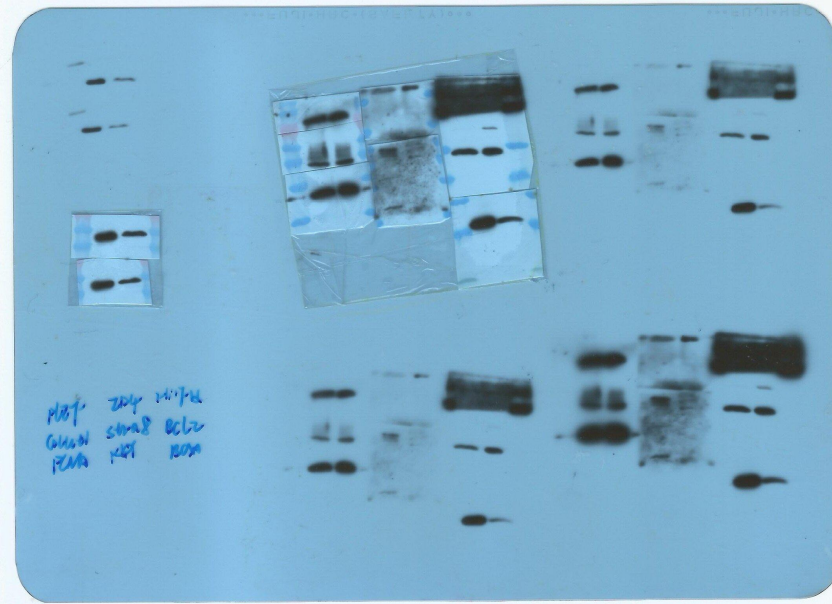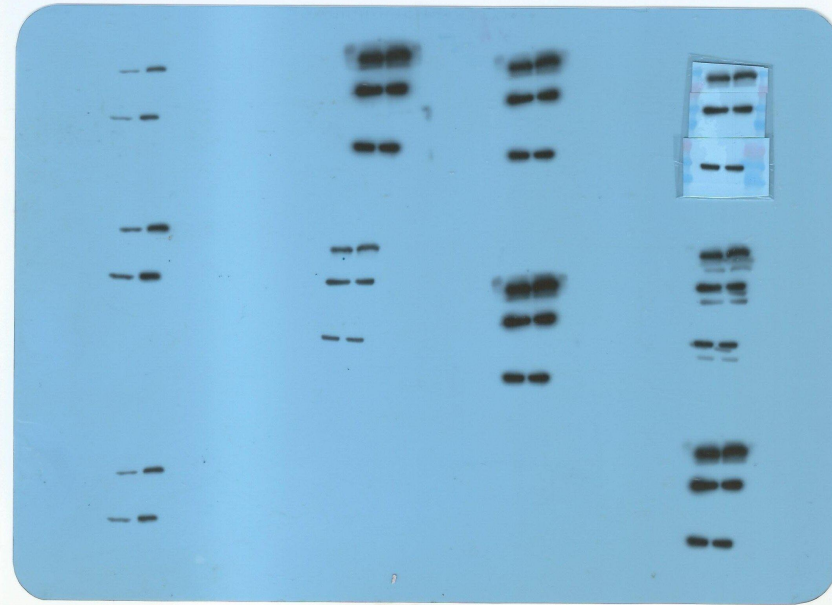

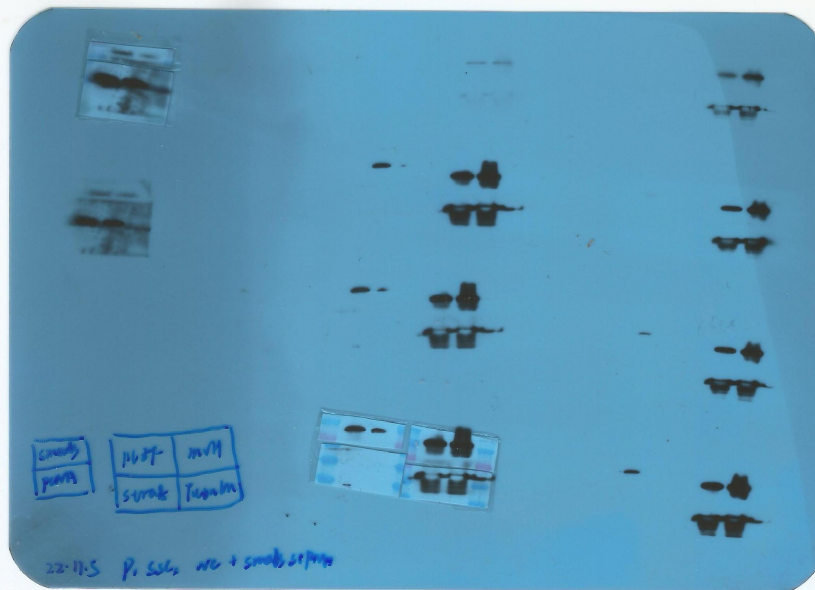

|         |      |      |
|---------|------|------|
| control | 1607 | 1611 |
| parent  | 1608 | 1612 |

22-11-5 P. 554 no + small 1607

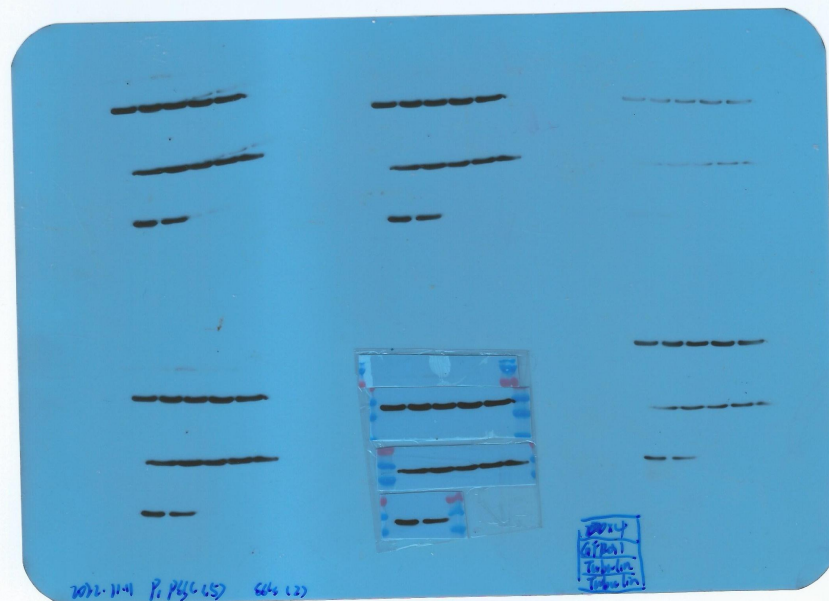

|         |      |      |
|---------|------|------|
| control | 1607 | 1611 |
| parent  | 1608 | 1612 |

2012-11-11 P. 746 (5) 604 (2)

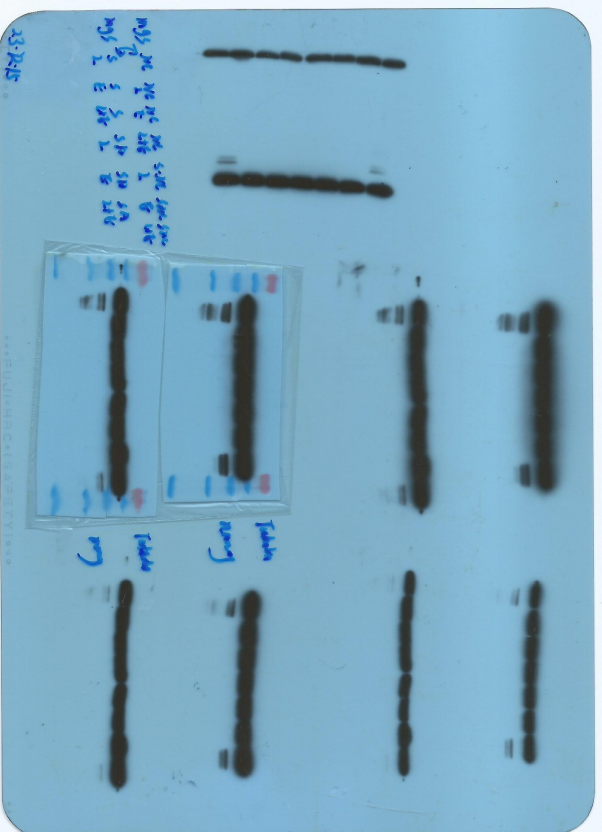

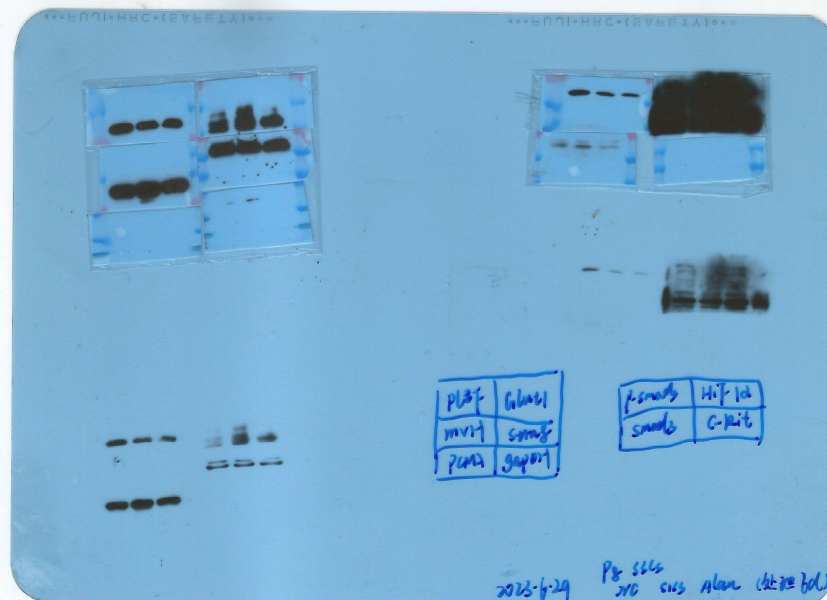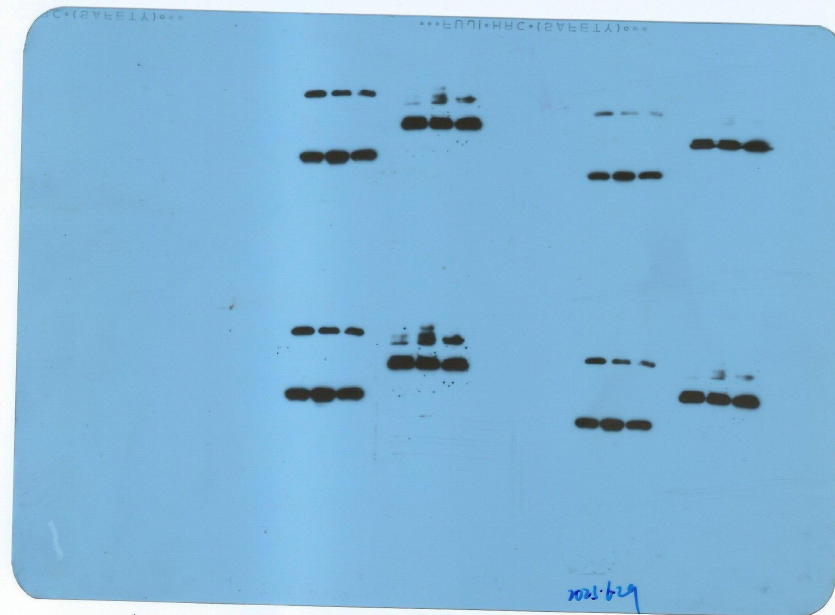

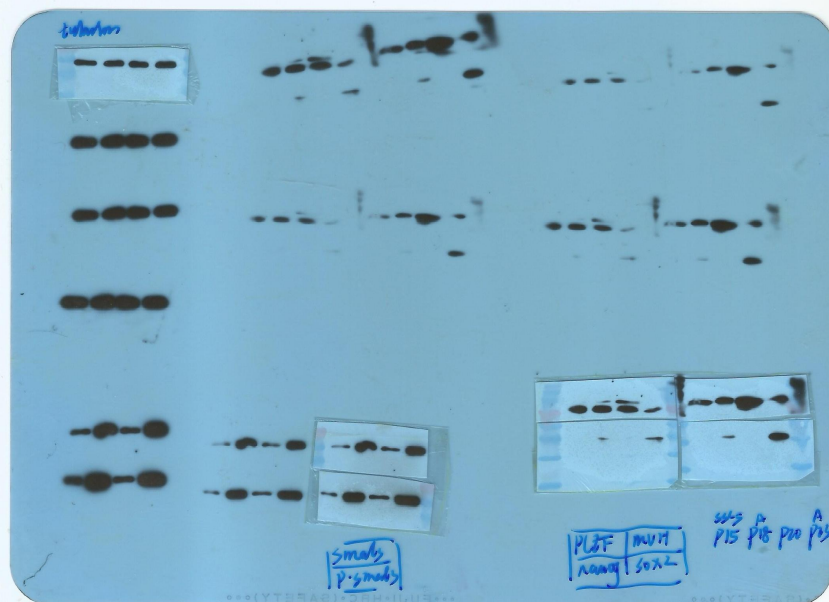

Supplement: Supplementary file 7 — Supplementary Material 7 [file 13287_2025_4541_MOESM7_ESM.pdf]
