# Supplementary material for: SMAD3 and HIF-1α orchestrate metabolic transition to aerobic glycolysis as a critical prerequisite for spontaneous reprogramming of spermatogonial stem cells
Source: Stem Cell Res Ther. 2025 Jul 28;16:411. doi: 10.1186/s13287-025-04541-w (PMC12305914; doi:10.1186/s13287-025-04541-w)
Supplement: Supplementary file 8 — Supplementary Material 8 [file 13287_2025_4541_MOESM8_ESM.docx]

**Figure S1. Confirmation of SSC identity through immunofluorescence assay.**

Antibodies against PLZF, GFRA1 and MVH were used for identification of SSCs, and dapi was used for counterstaining. Scale bar = 20 µm.

**Figure S2. The sample collection for metabolomics and detection of germline and pluripotent markers.**

The strategies of cell culture and sample collection (A). Principal component analysis score plot of mass spectrometry data of each group of samples (B). The changes of mRNA relative expression after treating SSCs with different conditions were detected by RT-PCR (C-E). The relative expression levels of key enzyme genes involved in glycolysis after increasing glucose concentration in culture system (C). The relative expression levels of SSCs reproductive and pluripotent markers after increasing glucose concentration in the culture system (D). The relative expression levels of SSCs reproductive system and pluripotent markers after adding glycolysis inhibitor 2-DG (E). The data represent the means ± SD (**p* < 0.05; **, *p* < 0.01).

**Figure S3. Differential expression analysis and enrichment analysis during pluripotent transformation of SSCs.**

1. PCA analysis of RNA-seq samples.(B) Volcano plots identifying differentially expressed genes between groups, where orange represents upregulated genes and yellow represents downregulated genes; Intermediate cells vs SSCs (top), GSPCs vs SSCs (middle), GSPCs vs Intermediate cells (bottom).(C) Heatmap of genes specifically expressed in GSPCs (right) and corresponding bar plot of GO functional enrichment analysis (left), with the black dashed line indicating the threshold p-value for enrichment analysis, and items above the dashed line having adjusted *p*-values <0.05.

**Figure S4. Methylation landscape during pluripotent transformation of SSCs.**

1. PCA Analysis of RRBS Sequencing Samples. (B) Annotation Pie Charts of Differentially Methylated Regions, including Genome Element Annotation (left) and CpG Region Annotation (right). (C) IGV Visualization of Methylation Sites in Pluripotency Marker Genes (*Klf4*, *Nanog*, *Myc*).

**Figure S5. Transcriptomic landscape of SSCs modulated by SMAD3 inhibition or activation.**

(A) PCA of the global gene expression profiles from SSCs post-SMAD3 intervention, with each point representing an individual biological replicate. (B) Comparative volcano plot highlighting gene expression alterations between NC and Alantolactone (Alan)-treated SSCs. (C) Volcano plot delineating the differential gene expression between control (NC) and SIS3-treated SSCs. (D) Upregulation of genes implicated in the inflammatory response within the Alan-treated cohort. (E) PPI network analysis demonstrating the association of SMAD3 with proteins involved in inflammatory processes.
